# Chiffon triggers global histone H3 acetylation and expression of developmental genes in *Drosophila* embryos

**DOI:** 10.1242/jcs.259132

**Published:** 2022-01-24

**Authors:** Eliana F. Torres-Zelada, Smitha George, Hannah R. Blum, Vikki M. Weake

**Affiliations:** 1 Department of Biochemistry, Purdue University, West Lafayette, IN 47907, USA; 2 Purdue University Center for Cancer Research, Purdue University, West Lafayette, IN 47907, USA

**Keywords:** Chiffon, *Drosophila*, Chromatin, Histone acetylation, Development, SAGA, CHAT

## Abstract

The histone acetyltransferase Gcn5 is critical for gene expression and development. In *Drosophila*, Gcn5 is part of four complexes (SAGA, ATAC, CHAT and ADA) that are essential for fly viability and have key roles in regulating gene expression. Here, we show that although the SAGA, ADA and CHAT complexes play redundant roles in embryonic gene expression, the insect-specific CHAT complex uniquely regulates expression of a subset of developmental genes. We also identify a substantial decrease in histone acetylation in *chiffon* mutant embryos that exceeds that observed in *Ada2b*, suggesting broader roles for Chiffon in regulating histone acetylation outside of the Gcn5 complexes. The *chiffon* gene encodes two independent polypeptides that nucleate formation of either the CHAT or Dbf4-dependent kinase (DDK) complexes. DDK includes the cell cycle kinase Cdc7, which is necessary for maternally driven DNA replication in the embryo. We identify a temporal switch between the expression of these *chiffon* gene products during a short window during the early nuclear cycles in embryos that correlates with the onset of zygotic genome activation, suggesting a potential role for CHAT in this process.

This article has an associated First Person interview with the first author of the paper.

## Abstract (precis)

**Summary:** Chiffon within the Gcn5-containing CHAT complex plays key roles in embryonic gene expression and histone H3 acetylation in *Drosophila*.

## INTRODUCTION

Histone acetylation stimulates chromatin remodeling, thereby contributing to transcription activation. One of the best-characterized histone acetyltransferases (HATs) is the highly conserved General control nonrepressed 5 (Gcn5), which functions as part of large multi-subunit transcriptional coactivator complexes to stimulate gene expression (Soffers and Workman, 2020). The fruit fly *Drosophila melanogaster* contains four Gcn5-containing complexes: Spt–Ada–Gcn5 acetyltransferase (SAGA) (Kusch et al., 2003; Muratoglu et al., 2003), Ada2a-containing complex (ATAC) (Guelman et al., 2006), Chiffon histone acetyltransferase (CHAT) (Torres-Zelada et al., 2019) and the Ada2–Gcn5–Ada3 transcription activator (ADA) (Soffers et al., 2019). The formation of each of these complexes is determined by which Ada2 homolog incorporates into the complex: ATAC contains Ada2a, while SAGA and ADA contain the Ada2b-PB splice isoform, and CHAT contains the Ada2b-PA isoform (Kusch et al., 2003; Muratoglu et al., 2003; Torres-Zelada et al., 2019; Weake et al., 2009). In general, all Gcn5 complexes preferentially acetylate histone H3 *in vitro* and *in vivo* with the highest activity on K9 and K14 (Guelman et al., 2006; Pankotai et al., 2005; Soffers et al., 2019; Torres-Zelada et al., 2019). In addition, ATAC acetylates histone H4 due to the presence of a second HAT within the complex (Suganuma et al., 2008). Mutations in *Ada2b* that disrupt SAGA, ADA and CHAT alter gene expression (Li et al., 2017; Weake et al., 2008). However, there is remarkably little overlap between the genes regulated by *Ada2b* and other SAGA subunits. Although this lack of overlap was previously attributed to SAGA's additional enzymatic activities, in light of findings that Ada2b splice isoforms nucleate formation of distinct Gcn5 complexes (Torres-Zelada et al., 2019), an alternative interpretation is that SAGA, ADA and CHAT have distinct roles in gene expression.

**Fig. 1.**
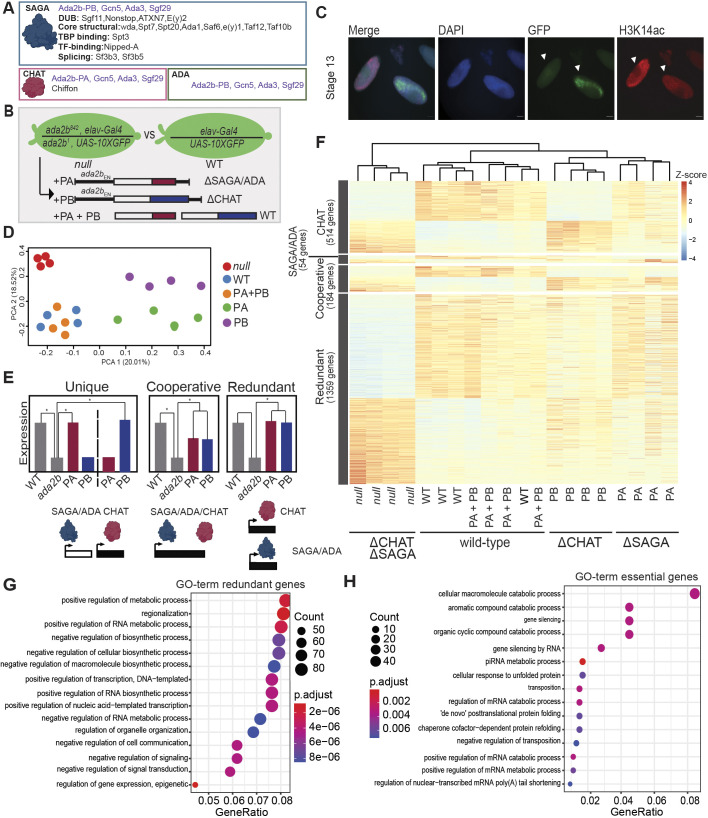
**Ada2b splice isoforms act redundantly to regulate gene expression in embryos.** (A) Schematic showing the differences in composition between the SAGA, ADA and CHAT complexes. The shared Gcn5 core subunits are labeled in purple. (B) Outline of the RNA-seq design. Flies that carry two different *Ada2b* null alleles (*Ada2b^842^* or *Ada2b^1^*) were crossed as outlined, and GFP-positive stage 12–14 embryos were manually selected. The Ada2b-PA or Ada2b-PB transgenes were expressed as single copies *in trans* under the control of the *Ada2b* genomic regulatory sequences (*Ada2b*_EN_). (C) Stage 13 embryos were stained for DAPI and H3K14ac. GFP-positive embryo is *Ada2b* null*.* Scale bars: 20 µm. (D) Scatterplot of principal component analysis (PCA) of normalized counts for each replicate. (E) Idealized bar plots demonstrating the criteria for Ada2b-regulated genes. * indicates difference as determined by statistical tests as detailed in text and methods. (F) Heatmap of RNA-seq expression *z*-scores computed for DEGs in *Ada2b* versus PA+PB and WT. (G,H) Gene ontology (GO) terms for genes regulated redundantly by SAGA/ADA and CHAT (G) or requiring CHAT for unique and cooperative expression (H).

CHAT contains three subunits that are shared with the other Gcn5 complexes, Gcn5, Ada3 and Sgf29, and two unique subunits, the Ada2b-PA splice isoform and Chiffon (Fig. 1A). In flies, *chiffon* encodes two polypeptides that have independent functions. The Chiffon N-terminal product is orthologous to Dbf4, which is a cyclin-like protein that binds and activates Cdc7 forming the Dbf4-dependent kinase (DDK) complex that initiates DNA replication (Landis and Tower, 1999; Stephenson et al., 2015); we refer to this polypeptide as Chiffon-A. In contrast, the C-terminal domain of Chiffon, which is only conserved within insects, directly binds Gcn5 and nucleates formation of the CHAT complex; we refer to this polypeptide as Chiffon-B. Only Chiffon-B is essential for fly viability because it rescues the lethal *chiffon* mutant phenotype, whereas Chiffon-A, which binds Cdc7, does not (Torres-Zelada et al., 2019). Intriguingly, CHAT can also substitute for SAGA or ADA HAT activity during fly development because expression of the CHAT-specific Ada2b-PA splice isoform restores viability to *Ada2b* mutants whereas the shared SAGA and ADA Ada2b-PB isoform does not (Torres-Zelada et al., 2019). These data suggest that either CHAT is the predominant HAT required for development in flies, or that CHAT can compensate for some of the functions of SAGA and/or ADA (hereafter SAGA/ADA) in acetylation (Torres-Zelada et al., 2019). To distinguish between these possibilities, we investigated the functional overlap between the SAGA, ADA and CHAT complexes in terms of gene regulation. Here, we show that the majority of genes with disrupted expression in *Ada2b* embryos are redundantly regulated by SAGA, ADA and CHAT. Surprisingly, *chiffon* mutants that disrupt only CHAT cause different changes in gene expression compared with loss of *Ada2b*, accompanied by a global loss of H3K14ac genome-wide in embryos. These data suggest that, in addition to its HAT activity, the Chiffon subunit of CHAT might have another role in gene expression that is independent of Gcn5. In addition, we identify a temporal switch between expression of the Chiffon-A and Chiffon-B polypeptides in early embryos that coincides with the second wave of zygotic transcription. We propose that CHAT functions as a pioneer coactivator complex during embryogenesis that is necessary for the later recruitment and/or activity of HAT complexes that activate gene expression programs essential for development.

## RESULTS

### SAGA/ADA and CHAT act redundantly to regulate gene expression in embryos

Gene expression profiling of *Ada2b* mutants has revealed widespread disruption of gene expression that was historically attributed to the loss of SAGA activity (Torres-Zelada and Weake, 2020). However, the recent finding that alternative splicing of *Ada2b* can generate new diversity in Gcn5 complexes raises the question of whether these complexes have overlapping or distinct roles in regulating gene expression. To answer this question, we generated *Ada2b* mutant embryos that express either the Ada2b-PA or Ada2b-PB isoform, resulting in embryos that lack CHAT, or SAGA and ADA, respectively (Fig. 1A,B). The Ada2b-PA or -PB transgenes were expressed *in trans* under control of the genomic *Ada2b* regulatory sequences (*Ada2b*_EN_), as described previously (Weake et al., 2011). The lethality observed in *Ada2b* mutants is entirely rescued by expression of both Ada2b splice isoforms (Torres-Zelada et al., 2019). We note that the SAGA and ADA complexes cannot be distinguished genetically in flies through *Ada2b* because the Ada2b-PB isoform is present in both complexes (Fig. 1A).

As wild-type (WT) controls, we used *Ada2b* embryos that express both Ada2b-PA and Ada2b-PB (which fully restore viability), as well as *­elav-Gal4>GFP* embryos that do not carry the *Ada2b* alleles (Fig. 1B). We observed a decrease in H3K14ac levels in the GFP-positive *Ada2b* null embryos relative to their heterozygous (non-GFP) siblings by stage 13, indicating that loss of all Ada2b isoforms globally impacts histone H3 acetylation (Fig. 1C). Based on these data, we performed RNA sequencing (RNA-seq) experiments in stage 12−14 embryos [8–11 h after egg laying (AEL)]. Principal component analysis (PCA) revealed that the *Ada2b* samples were distinct from the two WT controls (Fig. 1D). Interestingly, the *Ada2b* embryos expressing either the Ada2b-PA isoform (ΔSAGA/ADA) or the Ada2b-PB isoform (ΔCHAT) grouped more closely to each other rather than to either the null or WT samples. These data suggested that SAGA/ADA and CHAT might have redundant or overlapping roles in gene expression during embryogenesis.

To identify genes that required SAGA/ADA or CHAT for expression, we first identified genes that were differentially expressed between *Ada2b* and both WT controls. We identified 2111 differentially expressed genes (DEGs) between *Ada2b* and WT. We conclude that these 2111 DEGs, corresponding to 22% of all expressed genes, represent the complete set of gene targets for the Ada2 subunits of SAGA, ADA and CHAT in embryos (Table S1). Next, we reasoned that there were three distinct possibilities for how these Gcn5 complexes could regulate gene expression. If SAGA/ADA and CHAT regulate expression of unique sets of genes, then some of the 2111 DEGs would be misregulated in the same direction and to the same extent [fold change (FC)] in *Ada2b* embryos expressing either the Ada2b-PA or Ada2b-PB isoform (Fig. 1E). Alternatively, if SAGA/ADA and CHAT act cooperatively to regulate gene expression, then those DEGs would be misregulated in the same direction in both *Ada2b* embryos expressing Ada2b-PA (*Ada2b*+Ada2b-PA) and Ada2b-PB (*Ada2b*+Ada2b-PB), but to a lesser extent than the null. Last, if SAGA/ADA and CHAT act redundantly, then the identified DEGs would not be misexpressed in *Ada2b* embryos expressing either splice isoform because SAGA/ADA and CHAT would compensate for the loss of each other at these genes.

When we clustered the 2111 DEGs identified in *Ada2b* relative to WT, we found very few genes that were uniquely regulated by SAGA/ADA (*Ada2b*+Ada2b-PA, 54 genes) while nearly a quarter were uniquely regulated by CHAT (*Ada2b*+Ada2b-PB, 514 genes). Further, only 184 genes were cooperatively regulated by SAGA/ADA and CHAT, showing changes in expression upon loss of either isoform. In contrast, the majority of the DEGs (1359 genes) appear to be regulated redundantly by SAGA/ADA and CHAT, showing restored expression in the presence of either splice isoform (Fig. 1F). Examination of the normalized expression for these genes revealed that even at these redundantly regulated genes, loss of CHAT still had a stronger effect on gene expression relative to the loss of SAGA/ADA, particularly for downregulated genes. These data suggest that most genes that require SAGA, ADA or CHAT for proper expression are regulated redundantly by these complexes, with a slightly stronger role for CHAT relative to the other Gcn5 complexes in embryos.

Gene ontology (GO) analysis for these 1359 SAGA/ADA/CHAT-regulated genes showed enrichment for biological processes involved in development, such as regionalization, negative regulation of cell communication and positive regulation of transcription (Fig. 1G). Because Ada2b-PA is essential for fly viability but Ada2b-PB is not (Torres-Zelada et al., 2019), it is likely that either the 514 CHAT-regulated genes or the 184 SAGA/ADA/CHAT cooperatively regulated genes represent those critical genes that, when misexpressed at this embryonic stage, cause lethality later in development. For this group of genes, the most enriched GO terms included gene silencing, cellular macromolecule catabolic process and post-translational protein-folding processes (Fig. 1H). Although expression of Ada2b-PA restores adult viability to *Ada2b* mutants, only 63% of the expected adults emerge, suggesting that SAGA, ADA and CHAT act together to regulate the expression of genes that are essential for proper development. Overall, we conclude that SAGA/ADA and CHAT act redundantly at most genes in embryos, with a small proportion of genes being uniquely regulated by CHAT.

### CHAT is necessary for global H3K14ac in embryos

**Fig. 2.**
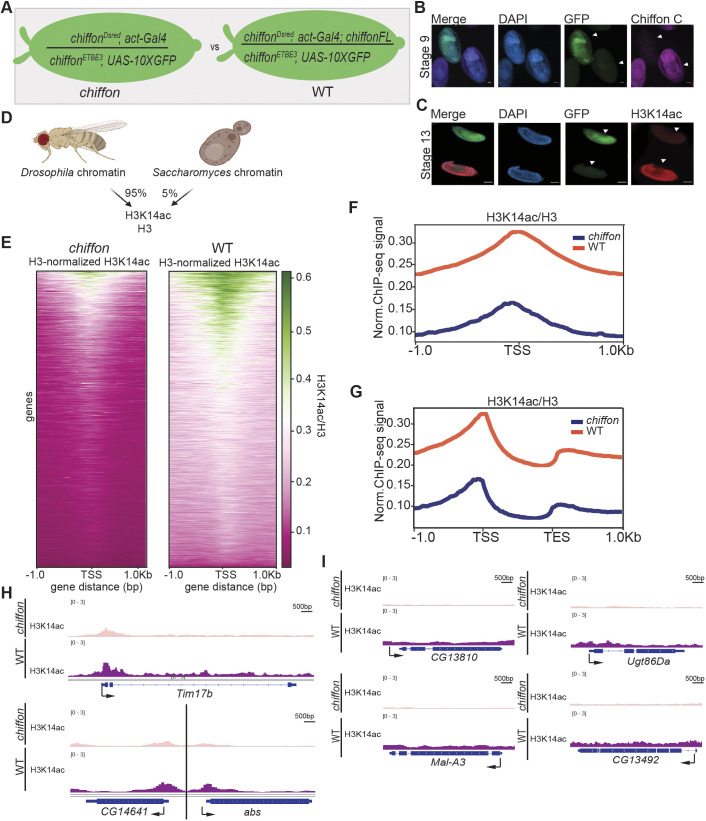
**CHAT is necessary for global H3K14ac levels in embryos.** (A) Outline of the ChIP-seq design. Stage 12–14 GFP-positive embryos that were *chiffon* null were compared with *chiffon* embryos expressing the Chiffon FL rescue transgene (WT). (B) Stage 9 embryos were stained with DAPI and an antibody raised against the C-terminal region of Chiffon (1400–1695 aa). GFP-positive embryo is *chiffon* null. Scale bars: 20 µm. (C) Stage 13 embryos were stained for DAPI and H3K14ac. (D) Schematic explaining spike-in normalization. Before starting ChIP, *Drosophila* chromatin was mixed with *Saccharomyces cerevisiae* chromatin in the indicated proportions. (E) Heatmaps showing reference-adjusted reads per million (RRPM)-normalized H3K14ac ChIP-seq signal around the transcription start site (TSS) of protein-coding genes in *chiffon* and WT embryos. (F) Metaplot of RRPM-normalized H3K14ac ChIP-seq signal around the TSS averaged for all protein-coding genes in *chiffon* (blue) and WT (orange). (G) Metaplots of *chiffon* (blue) and WT (orange) RRPM-normalized H3K14ac ChIP-seq signal over gene bodies averaged for all protein-coding genes. (H) Genome browser inspection using Integrative Genomics Viewer (IGV) of RRPM-normalized H3K14ac signal at representative genes comparing *chiffon* and WT. Data scaled to the same height for all comparisons (0–3). (I) Genome browser snapshots (IGV) showing RRPM-normalized H3K14ac signal for three representative genes containing unique H3K14ac peaks for *chiffon* and WT samples.

Because SAGA/ADA and CHAT shared overlapping roles in gene expression in embryos, we next asked how loss of CHAT affected histone acetylation in embryos. We previously showed that CHAT acetylates histone H3 with specificity for lysines 9, 14 and 18, with *chiffon* null ovary follicle cells showing a 50% decrease in H3K14ac levels (Torres-Zelada et al., 2019). We used a similar genetic approach to that used for *Ada2b* to positively label embryos that contained two different *chiffon* null alleles with GFP (Fig. 2A). Using an antibody raised against the unique C-terminal region of Chiffon [1400–1695 amino acids (aa)], we showed that GFP-positive *chiffon* embryos have a substantial decrease in Chiffon protein levels by stage 9 (Fig. 2B). Surprisingly, when we examined H3K14ac levels in *chiffon* embryos, we observed a stronger decrease in H3K14ac than that observed in *Ada2b* mutants that disrupt SAGA, ADA and CHAT (compare Fig. 2C and Fig. 1C; Fig. S1A,B). When we quantified these data, we found that *chiffon* embryos showed 40% of the H3K14ac signal relative to their heterozygote siblings, versus 70% in *Ada2b* embryos (Fig. S1C). We also observed a significant decrease in H3K18ac in GFP-positive *chiffon* embryos when compared with their heterozygous siblings, however to a lesser extent than H3K14ac (Fig. S1D,E). These data show that loss of *chiffon* substantially decreases H3K14ac levels in embryos.

Because Ada2 subunits are essential for the nucleosomal HAT activity of Gcn5 (Grant et al., 1997), we next asked whether Ada2b-PA, Gcn5, Sgf29 and Ada3 could associate in the absence of Chiffon. To do this, we expressed each subunit in Sf21 cells using Baculovirus, and tested for direct interaction by co-immunoprecipitation (Fig. S2). Using this approach, we found that Ada2b-PA directly binds Gcn5 and Ada3, but not Sgf29, which is recruited instead through Ada3. Because Ada2b-PA is necessary and sufficient for Gcn5, Sgf29 and Ada3 to associate even in the absence of Chiffon, we would therefore expect that loss of *Ada2b* would disrupt CHAT formation. Thus, the loss of Chiffon in embryos has a stronger impact on histone acetylation that can be explained simply by loss of SAGA, ADA and CHAT HAT activity. Moreover, the decrease in H3K14ac in *chiffon* embryos differs from our previous observations in ovary follicle cells in which *chiffon* and *Ada2b* mutants both showed ∼50% decreases in H3K14ac relative to their respective controls (Torres-Zelada et al., 2019).

To investigate how loss of *chiffon* affects H3K14ac genome-wide, we next performed H3K14ac chromatin immunoprecipitation (ChIP) followed by Illumina sequencing (ChIP-seq) in GFP-positive *chiffon* stage 12–14 embryos (8–11 h AEL). As a WT control, we performed ChIP-seq in *chiffon* embryos expressing the full-length Chiffon rescue transgene (Chiffon FL), which restores viability and H3K14ac levels (Torres-Zelada et al., 2019). The Chiffon FL transgene was expressed *in trans*, as for Ada2b-PA or -PB (Fig. 1B), under control of the *chiffon* genomic regulatory sequences. We examined H3K14ac levels relative to histone H3 to control for differences in nucleosomal occupancy, and sequenced input chromatin controls for each sample. Because we suspected that loss of *chiffon* would result in a global decrease in H3K14ac levels based on our observations from the embryo immunostaining, we included spike-in *Saccharomyces cerevisiae* chromatin, enabling us to normalize H3K14ac signal to this internal control (Fig. 2D; Table S3). We then compared spike-in-normalized H3K14ac levels relative to histone H3 around the transcription start site (TSS) of all genes in *chiffon* embryos relative to WT (Fig. 2E,F). We observed a striking decrease in H3K14ac in *chiffon* embryos that was also readily observed in the individual three biological replicates (Fig. S3). Although H3K14ac is predominantly associated with promoters, a recent study has demonstrated peaks of H3K14ac in gene bodies, most likely due to the activity of the HAT Chameau rather than Gcn5 (Regadas et al., 2021). Our data also showed a decrease in H3K14ac gene body peaks in *chiffon* embryos both globally and at representative genes (Fig. 2G,H). Strikingly, many of the H3K14ac peaks that are present at genes that lack canonical histone acetylation (and are thought to be deposited by Chameau) are also lost in *chiffon* embryos (Fig. 2I). Taken together, these data argue that, rather than specifically reducing H3K14ac levels only at CHAT-regulated genes, loss of the *chiffon* subunit in CHAT leads to a decrease in global levels of H3K14ac in embryos by affecting the activity and/or recruitment of other HATs.

### Chiffon regulates expression of genes expressed in mid/late embryogenesis

**Fig. 3.**
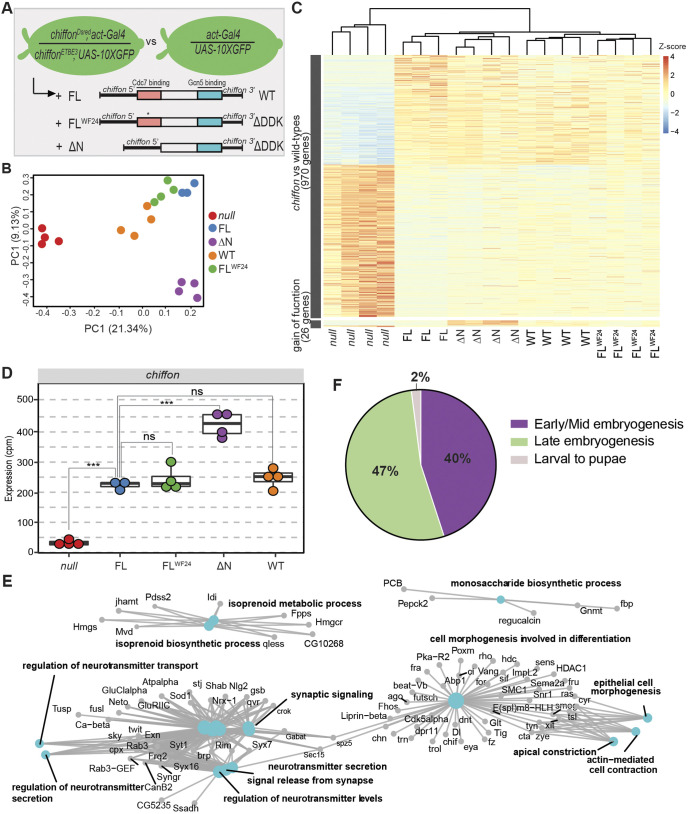
**Chiffon regulates gene expression in embryos.** (A) Outline of the Chiffon RNA-seq design. Flies that carry two different *chiffon* null alleles (*chiffon^ETBE3^ or chiffon^Dsred^*) were crossed as outlined, and GFP-positive stage 12–14 embryos were manually selected as *chiffon* null. Chiffon rescue transgenes were expressed *in trans* under control of their indicated *chiffon* 5′ and 3′ regulatory regions (black boxes). (B) PCA of normalized counts for each sample. (C) Heatmap of RNA-seq expression *z*-scores computed for DEGs in *chiffon* versus FL and WT. (D) Box plot showing counts per million (cpm) of replicate normalized counts for *chiffon* across all genotypes and samples. Box plots with overlaid points were generated using ggplot2; lower and upper hinges correspond to the first and third quartiles, and the whiskers extend to the smallest or largest values no more than 1.5x inter-quartile range (IQR) from each hinge. *P-*values for the indicated comparisons were determined by one-way ANOVA+Dunnett; ***P<0.0001; ns, not significant. (E) Gene Concept Network plot (Cnetplot) highlighting linkage of individual genes and associated functional categories of over-represented genes in *chiffon* embryos. (F) Proportion of CHAT-regulated genes in each developmental cluster.

Because *chiffon* embryos showed a more substantial decrease in H3K14ac relative to *Ada2b*, suggesting that the Chiffon subunit within CHAT functions upstream of other HATs, we next asked how loss of *chiffon* impacted gene expression in embryos. To do this, we performed RNA-seq in *chiffon* embryos at stage 12–14 (8–11 h AEL) as outlined in Fig. 3A. As WT controls, we used either *chiffon* embryos that express a single copy of a Chiffon FL transgene or the parental *act-Gal4>GFP* embryos (Fig. 3A). Expression of Chiffon FL restores viability to *chiffon* mutants (Torres-Zelada et al., 2019). We identified 996 genes that were differentially expressed between *chiffon* and both WT controls (Table S2). Because *chiffon* encodes independent Chiffon-A and Chiffon-B polypeptides that nucleate formation of DDK and CHAT, respectively (Torres-Zelada et al., 2019), the 996 DEGs identified in *chiffon* embryos could represent targets for either DDK or CHAT activity. Because expression of the ΔN-terminal transgene that restores CHAT function rescues both H3K14ac and adult viability in *chiffon* mutants (Torres-Zelada et al., 2019), we hypothesized that CHAT is necessary for histone H3 acetylation and gene expression in embryos. However, because DDK phosphorylates histone H3T45 in yeast and mammalian cells (Baker et al., 2010), it is possible that DDK also contributes to gene expression. To distinguish between these possibilities, we performed RNA-seq on *chiffon* mutants that express single copies of each of the following transgenes: ΔN-terminal (ΔN; 401–1695 aa) and FL with a stop codon at position 174, corresponding to the previously identified Chiffon FL^WF24^ allele (Fig. 3A) (Landis and Tower, 1999). The ΔN and FL^WF24^ transgenes restore viability to *chiffon* mutants because they express Chiffon-B and rescue CHAT function. However, the ΔN and FL^WF24^ constructs do not express Chiffon-A and do not restore DDK activity; hence, the resulting adult females are sterile due to lack of gene amplification in ovary follicle cells (Torres-Zelada et al., 2019).

PCA revealed that the *chiffon* samples were distinct from the two WT controls and the FL^WF24^ genotype, which grouped together (Fig. 3B). These data suggested that the FL^WF24^ embryos that lack DDK activity were most similar to WT embryos rather than *chiffon* mutants, indicating that the loss of CHAT activity is responsible for most of the differences in gene expression observed in the *chiffon* embryos. Moreover, examination of relative gene expression levels at the 996 DEGs in each genotype revealed that the ΔN and FL^WF24^ genotypes largely resembled the WT controls (Fig. 3C). These data indicate that all of the 996 DEGs identified in *chiffon* embryos are regulated by CHAT rather than DDK. Interestingly, the ΔN transgene rescue construct was separated along the second PC from both the WT and FL^WF24^ samples (Fig. 3B), suggesting that although the ΔN and FL^WF24^ transgenes both restore CHAT function, their activity differs. The ΔN and FL^WF24^ transgenes produce the same ∼48 kDa Chiffon-B protein product that binds Gcn5 and nucleates CHAT formation (Torres-Zelada et al., 2019), but differ at the nucleic acid level because the ΔN transgene lacks the first 1200 bp of the *chiffon* coding region. In addition, whereas the FL^WF24^ transgene fully restores viability to *chiffon* mutants, only 66% of the expected adults emerged in *chiffon* mutants expressing the ΔN transgene, suggesting that these 1200 bp of *chiffon* might contribute to the proper expression of CHAT (Torres-Zelada et al., 2019). We observed significantly higher expression of the ΔN transgene relative to the other constructs (Fig. 3D), suggesting that this 1200 bp region contains negative regulatory elements that control *chiffon* transcript levels. If so, the higher expression of the ΔN transgene relative to FL^WF24^ could result in a slight gain of function for CHAT activity in terms of gene expression. Supporting this, 26 genes were differentially expressed between the ΔN and FL^WF24^ genotypes, showing an opposite direction compared to the *chiffon* null (Fig. 3C; Table S2). Taken together, these data demonstrate that the Chiffon subunit with CHAT is necessary for expression of 10% of expressed genes in *Drosophila* embryos, with no detectable contribution from the DDK complex to gene expression at this developmental stage.

GO term analysis revealed that the *chiffon* DEGs were enriched for biological processes including isoprenoid metabolic processes, regulation of neurotransmitters and cell morphogenesis involved in differentiation (Fig. 3E). Moreover, the 427 downregulated genes (Fig. S4A), which represent potential targets for transcription activation by CHAT, were enriched for GO terms including salivary gland morphogenesis, ecdysone biosynthetic process and cell morphogenesis involved in gastrulation (Fig. S4B). Based on the enrichment of terms involved in development, we wondered whether CHAT regulates developmental genes that first initiate expression during late embryogenesis. To examine this, we compared CHAT-regulated genes with published developmental gene clusters (Graveley et al., 2011). Although only 57% of the CHAT-regulated genes (570/996) fall into any of these developmental expression clusters, of these genes, 40% (228/570) are strongly associated with early-to-mid embryogenesis while another 47% (271/570) are associated with late embryogenesis and larvae stage (Fig. 3F). Overall, our studies demonstrated that Chiffon regulates the expression of genes induced during embryo development.

Surprisingly, the overlap between the DEGs identified in the *Ada2b* and *chiffon* mutants is quite low: 213 genes (Fig. S4C). GO categories for these 213 genes include post-translational protein folding, positive regulation of cell cycle process and polytene chromosome puffing (Fig. S4D). Based on these observations, and the stronger decrease in H3K14ac in *chiffon* embryos relative to *Ada2b*, we conclude that the Chiffon-B subunit within the CHAT complex regulates gene expression in part through recruiting Gcn5 to chromatin to acetylate histone H3. However, our data suggest that Chiffon-B has additional roles in gene expression that are distinct from Ada2b/Gcn5 within CHAT, potentially functioning as a transcription coactivator. Our data further suggest that Chiffon-B activity is necessary for the recruitment and/or activity of other HAT complexes that target histone H3.

### A switch between expression of the DDK and CHAT Chiffon products during embryonic development triggers CHAT formation prior to cellularization

Because *chiffon* encodes two independent polypeptides that nucleate DDK or CHAT complex formation, and because loss of DDK activity had little effect on gene expression in late-stage embryos and was dispensable for viability in flies (Torres-Zelada et al., 2019), we wondered whether the Chiffon-A product that binds Cdc7 was even expressed in embryos. In *Drosophila* embryos, the first 13 cell cycles are maternally programmed and occur synchronously with extremely short cycles that exhibit no gap phases. Cdc7 is essential for these early embryonic cell cycles, and its protein signal declines by nuclear cycle (NC) 14 when the mid-blastula transition (MBT) initiates (Seller and O'Farrell, 2018). However, it was unclear whether Cdc7 requires Chiffon-A for its activity during early embryogenesis because Chiffon-A is entirely dispensable for adult viability, whereas Cdc7 is an essential gene (Stephenson et al., 2015).

**Fig. 4.**
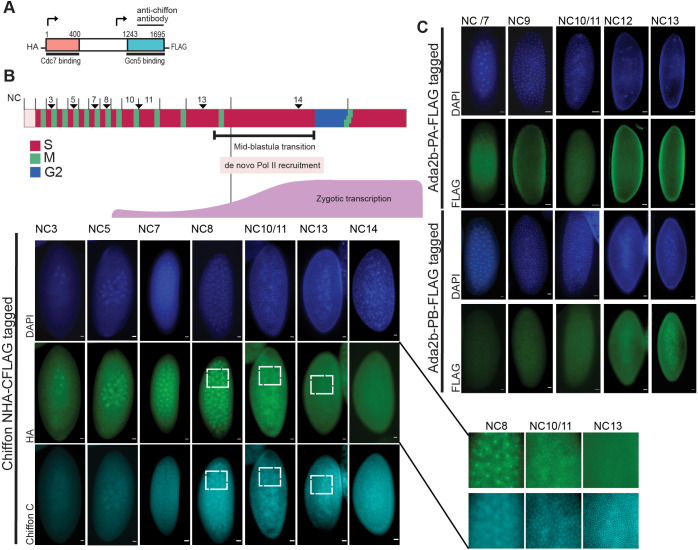
**The DDK and CHAT complexes are expressed sequentially during the early nuclear embryonic cycles*.*** (A) Schematic of the epitope-tagged FL Chiffon transgene. Chiffon transgene is HA tagged in the N-terminal domain and FLAG tagged in the C-terminal domain. (B) Diagram highlighting key events that occur during the maternally driven embryo nuclear cycles (NCs). There is an initial wave of Pol II recruitment and zygotic transcription at NC8 followed by a second more widespread wave of Pol II recruitment and transcription at NC13. Immunostaining of embryos at the indicated NC with HA and anti-Chiffon. Embryos were co-stained with DAPI and staged according to the number/position of nuclei. Adjacent insets: enlarged views of highlighted areas. Representative images are shown (*n*≥3). (C) Immunostaining of embryos during the indicated NC with FLAG to detect Ada2b-PA or Ada2b-PB. Embryos were co-stained with DAPI to visualize nuclei. Scale bars: 20 µm.

To address this issue, we generated an epitope-tagged full-length *chiffon* transgene that was HA tagged on its N-terminus, and expressed this as the sole copy of Chiffon *in trans* in flies carrying two *chiffon* null alleles. To assess the expression of Chiffon-A and -B, we then co-stained embryos with anti-HA antibodies to detect Chiffon-A containing the Cdc7-binding domain (DDK complex), together with an anti-Chiffon antibody raised against the C-terminal end of Chiffon to detect Chiffon-B (CHAT complex) (Fig. 4A). No background immunostaining signal was detected in untagged WT embryo (*w^1118^*) immunostained for HA under the conditions used (Fig. S5A). In addition, the anti-Chiffon antibody is specific for Chiffon-B because we do not detect Chiffon signal in the GFP-positive *chiffon* mutant embryos immunostained with this antibody (Fig. 2B). We determined the NC of each embryo by examining nuclei number using 4′,6-diamidino-2-phenylindole (DAPI) staining. We observed HA signal corresponding to Chiffon-A expression from NC3 to NC14, in a pattern resembling the published expression pattern of Cdc7 (Seller and O'Farrell, 2018) (Fig. 4B). However, consistent with the lack of gene expression defects in later-stage embryos lacking DDK, we did not detect expression of Chiffon-A (HA) after NC11 with no detectable expression in later-stage embryos (Fig. 4B, Fig. S5B). In contrast to the early embryonic expression of Chiffon-A, we did not detect expression of the Chiffon-B (anti-Chiffon) until NC10/11, after which we observed continued expression throughout the later stages of embryogenesis. Notably, both Chiffon-A and -B were detected together only at NC10/11 (Fig. 4B), suggesting that Chiffon FL might be present only transiently, if at all, at these NCs. Even if full-length Chiffon does exist transiently during NC10/11, our previous studies suggest that the full-length protein does not have an essential role (Torres-Zelada et al., 2019).

**Fig. 5.**
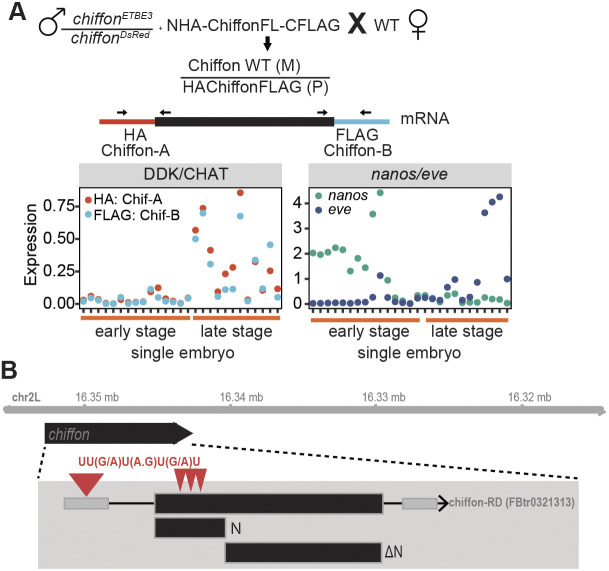
**The switch between Chiffon-A and Chiffon-B expression is not regulated at the mRNA level.** (A) Male flies expressing the epitope-tagged Chiffon FL transgene were crossed to untagged females (*w^1118^*), and expression of the paternal *chiffon* gene was assessed in single embryos using qRT-PCR with primers specific for Chiffon-A (using HA forward primer and *chiffon* 5′ reverse primer) or Chiffon-B (using a *chiffon* forward primer in the 3′ region and a FLAG reverse primer). Transcript levels were measured in single embryos and are shown relative to *RpL32*. Single embryos were ranked using the ratio of expression of *nanos* (*nos*; early) versus *even skipped* (*eve*; late) to provide a relative indication of early versus later developmental stage. Each dot represents a single relative transcript measurement (e.g. *chiffon-A*/*RpL32*) for one individual embryo, so that relative levels of Chiffon-A and Chiffon-B mRNA can be compared directly. (B) Schematic of *chiffon* gene showing Bruno response elements present in the first 1200 bp (N) that are absent from ΔN region.

Chiffon-A and Chiffon-B are both encoded from a single, large ∼5 kb exon in the *chiffon* gene, and Northern blotting analysis identified a single 6.5 kb *chiffon* transcript in *Drosophila* embryos (Landis and Tower, 1999), arguing against the presence of alternative splice isoforms. To further test whether there were differences in Chiffon-A or Chiffon-B expression at the mRNA level, we performed quantitative real-time PCR (qRT-PCR) on single embryos with primers that specifically detected Chiffon-A (5′ product) or Chiffon-B (3′ product) (Fig. 5A). To provide a relative indication of stage, we ranked single embryos by the ratio of expression of *nanos* (*nos*) and *even skipped* (*eve*), which are expressed early or late during the NCs, respectively (Thomsen et al., 2010). Using this approach, we did not identify any substantial differences in the relative expression of Chiffon-A and Chiffon-B at the mRNA level, suggesting that the full-length transcript is present throughout these stages of embryogenesis. These data suggest that the switch between Chiffon-A and Chiffon-B expression is not controlled by alternative splicing and may involve translational mechanisms (Fig. 5B, see Discussion).

Our data suggest that there is a switch at NC10/11 between the expression of the Chiffon-A product that nucleates DDK formation and the Chiffon-B product that nucleates CHAT formation. We wondered, therefore, whether expression of this Chiffon-B product coincided with the recruitment of Ada2b-PA within CHAT to the nucleus. To test this, we used anti-FLAG antibodies to immunostain embryos expressing epitope-tagged Ada2b-PA. No background immunostaining signal was detected in untagged WT embryo (*w^1118^*) immunostained for FLAG under the conditions used (Fig. S5C). Similar to the nuclear localization of Chiffon-B starting at NC10/11, Ada2b-PA showed nuclear-localized staining beginning at NC10/11 (Fig. 4C). Intriguingly, Ada2b-PB also showed a similar pattern of immunostaining beginning at NC10/11, albeit with weaker signal intensity, suggesting that SAGA/ADA recruitment to the nucleus occurs during the same temporal window as CHAT recruitment.

### Chiffon-A within DDK is maternally required for early embryonic development

**Fig. 6.**
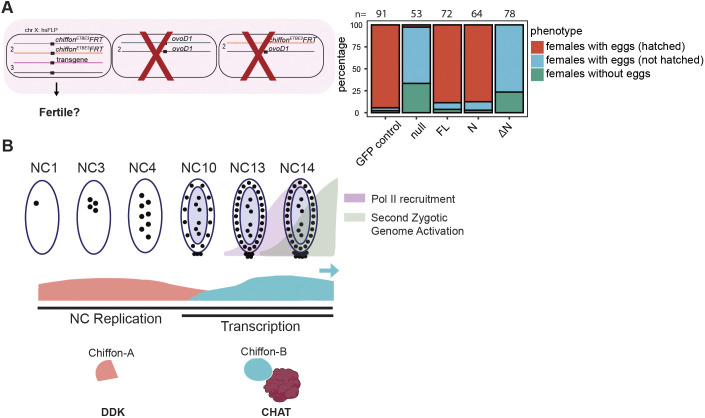
**DDK and CHAT complexes have distinct roles in early embryo development.** (A) Germline clones for *chiffon* were generated to assess whether DDK or CHAT function was necessary for early embryonic development. Fertility was examined in individual females (*n* shown above each bar). (B) Model illustrating the developmental switch between expression of the Chiffon-A and Chiffon-B products that nucleate formation of DDK or CHAT, respectively. The expression of Chiffon-A is highest during the early NCs when DNA replication occurs rapidly, and starts to diminish as the NCs slow and zygotic transcription begins. The onset of Chiffon-B expression occurs just prior to the second wave of zygotic transcription, suggesting a potential role in this process.

We previously showed that the DDK activity of Chiffon was not necessary for adult viability, but was essential for follicle cell amplification (Torres-Zelada et al., 2019). Because *Cdc7* is necessary for DNA replication during the early cell cycles (Seller and O'Farrell, 2018), we hypothesized that the DDK activity of *chiffon* (i.e. Chiffon-A) is also required maternally for embryo development. To test this, we generated germline mosaic clones in which *chiffon* was maternally depleted (Fig. 6A). Loss of *chiffon* results in a complete failure of embryos to hatch, suggesting that *chiffon* is required maternally for embryo development but is not essential for oogenesis (Fig. 6A). We could rescue this hatch defect by expressing either the FL or N-terminal Chiffon transgenes, suggesting that restoring DDK activity is sufficient for Chiffon's function in these early nuclear cell cycles. In contrast, the Chiffon ΔN transgene did not restore embryo hatching (Fig. 6A), suggesting that maternal CHAT is not necessary for these early stages of embryonic development.

Altogether, our data show that Chiffon-B nucleates the formation of CHAT in the early NCs of *Drosophila* embryogenesis before cellularization. We propose that this early formation of CHAT triggers histone H3 acetylation, and is necessary for the subsequent recruitment and/or activity of other histone H3 HATs such as SAGA, ADA, and even Nejire or Chameau. Alternatively, Chiffon-B might have additional functions in controlling gene expression and histone acetylation outside of the CHAT complex. Notably, CHAT formation occurs just prior to the *de novo* large-scale recruitment of RNA polymerase II (NC13–14) that leads to the second and massive wave of the zygotic genome (Chen et al., 2013), suggesting that the timing of CHAT formation could have a key role in activating this wave of early transcription (Fig. 6B).

## DISCUSSION

Our data support a widespread and overlapping role for the three Ada2b-containing Gcn5 complexes, SAGA, ADA and CHAT, in embryonic gene expression in *Drosophila*. Consistent with the essential role of Ada2b-PA in fly viability, loss of Ada2b-PA resulted in a stronger effect on gene expression relative to Ada2b-PB, suggesting that CHAT uniquely regulates nearly a quarter of the Ada2b-dependent genes in embryos. Surprisingly, we observed stronger loss of histone acetylation in *chiffon* mutant embryos relative to *Ada2b*, coupled with largely non-overlapping changes in gene expression in *chiffon* versus *Ada2b* mutant embryos. These data suggest that Chiffon might have functions outside of the CHAT complex with regards to both gene regulation and histone acetylation. However, our previous mass spectrometry analysis of Chiffon-B-interacting proteins in *Drosophila* S2 cells did not identify any HATs other than Gcn5 (Torres-Zelada et al., 2019). It is possible that any interactions between Chiffon-B and other HATs might be unique to embryos because we did not observe the same level of H3K14ac decrease in *chiffon* mutant ovary follicle cells (Torres-Zelada et al., 2019). Moreover, a much higher number of transcription factors was identified in mass spectrometry of SAGA purified from embryos versus S2 cells (Weake et al., 2011), suggesting that many protein interactions may only be detected in the relevant tissue or developmental stage. Supporting a potential connection between Chiffon and other histone H3 HATs, *chiffon* embryos showed loss of many of the H3K14ac peaks that are present at genes that lack canonical histone acetylation (H3K14ac unique peaks) and are thought to be deposited by Chameau (Regadas et al., 2021). We recognize that identifying the direct gene targets of Chiffon-B in embryos is critical to provide insight into whether Chiffon-B has roles outside of CHAT; however, our efforts to characterize the genome-wide distribution of the CHAT complex have been technically challenging, potentially due to the presence of only a single FLAG tag on Chiffon-B, which has resulted in inefficient ChIP in our hands. Other groups have also reported technical difficulties in obtaining reliable ChIP-seq profiles for many of the Gcn5-containing complexes (Fischer et al., 2021).

Intriguingly, expression of the Chiffon-B polypeptide that nucleates CHAT formation is first detected around NC11, just prior to the second and large-scale recruitment of RNA polymerase II (Pol II) that leads to major activation of the zygotic genome (Chen et al., 2013) (Figs. 4B and 6B). Because the timing of CHAT formation just precedes zygotic genome activation, we propose that CHAT might have a critical role in regulating the timing of this process, albeit potentially redundant with other coactivator complexes. During *Drosophila* embryo development, one key player that activates the genome in the early embryo is the pioneer transcription factor Zelda (Liang et al., 2008). Zelda binds its target genes as early as NC8, when the earliest wave of zygotic transcription occurs (Li et al., 2014; Pritchard and Schubiger, 1996). There is a high degree of overlap between histone acetylation at H3K18 and K27, H4K8, and nucleosome remodeling around Zelda binding sites (Li et al., 2014), suggesting that HAT activity contributes to Zelda-mediated gene activation. Notably, ChIP-seq analysis shows that these histone marks are all enriched at NC8 and continue to increase substantially in levels through NC14 (Li et al., 2014). Nejire (CBP-p300) is regarded as one of the major HATs that contributes to these particular histone acetyl marks (Tie et al., 2009), suggesting that it might be the major HAT that functions during the early first wave of zygotic transcription. However, Zelda might require additional interacting partners at NC14 that are only expressed just prior to this stage (Harrison et al., 2011); we propose that Chiffon-B, as part of CHAT, could constitute a key partner for Zelda in this second wave of zygotic genome activation. Notably, H3K14ac was not examined in the study that identified increases in the other histone acetyl marks that correlate with the first and second waves of zygotic transcription (Li et al., 2014), so it remains possible that Chiffon-B and CHAT could play an as-yet unrecognized role in this process.

What could be responsible for the switch in expression between Chiffon-A and Chiffon-B during early embryonic development? Translational control of maternally deposited mRNAs plays a central role in early *Drosophila* development because the two waves of zygotic transcription do not begin until NC8 and NC14 (Chekulaeva et al., 2006; Hamm and Harrison, 2018; Tadros and Lipshitz, 2009). Many RNA-binding proteins control translation of mRNAs; for example, the *Drosophila* RNA-binding protein Bruno (also known as Bruno 1) binds to specific Bruno response elements (BREs), inhibiting translation of these BRE-containing mRNAs (Chekulaeva et al., 2006; Tarn and Lai, 2011). Interestingly, there are three BREs in the Chiffon-A region of the *chiffon* mRNA that could negatively regulate the translation of Chiffon N region after NC11 (Fig. 5B). Supporting the hypothesis that negative regulatory elements are present in this N-terminal region that spans ∼1200 bp, the ΔN transgene that lacked this region showed partial gain-of-function effects with respect to CHAT gene expression activity. We propose that the unique dicistronic gene structure of *chiffon* allows it to act as a developmental switch to trigger the timing of zygotic genome activation at the same time that embryonic NCs start to slow, due in part to decreased DDK activity (Fig. 6B). Because the C-terminal extension of Chiffon is only conserved within insect orthologs of Dbf4, it is likely that other mechanisms play a role in this transition in vertebrates and other animals.

## MATERIALS AND METHODS

### Genetics

Flies were raised in a 12:12 h light:dark cycle at 25°C on standard fly food (Lewis, 1960). Genotypes for flies used in this study are described in Table S4. For RNA-seq experiments, flies were generated carrying two different *chiffon* (*chiffon^ETBE3^* and *chiffon^DsRed^*) (Torres-Zelada et al., 2019) or *Ada2b* (*Ada2b^1^* and *Ada2b^842^*) (Pankotai et al., 2005, 2013) null alleles on chromosome 2 (*chiffon*) or chromosome 3 (*Ada2b*), respectively, as either *Actin-Gal4* (or *elav-Gal4* for *Ada2b*) or *UAS-10XGFP*. To identify homozygous *chiffon* or *Ada2b* mutants, we crossed flies as outlined in Figs 1 and 3 and manually selected GFP-positive embryos that carry the two different null alleles. Chiffon rescue transgenes contain genomic *chiffon* enhancer sequences that span −3480 bp relative to the translation start site and include the *chiffon* 3′ UTR sequences that extend 1056 bp past the stop codon as described in Torres-Zelada et al. (2019). Ada2b transgenes were expressed *in trans* under control of their genomic *Ada2b* enhancer sequences (*ada2b*_EN_) that begin −1878 bp from the TSS and extend +1782 bp to the end of the second exon, as described in Weake et al. (2011). The Chiffon C-terminal transgene previously referred to as Chiffon-C in Torres-Zelada et al. (2019) is referred to as ΔN in this study. For ChIP-seq experiments, homozygous *chiffon* mutants or WT control were selected as outlined in Fig. 3. We generated an epitope-tagged full-length *chiffon* transgene that was HA tagged on its N-terminal domain, and FLAG tagged on its C-terminal domain, and expressed this as the sole copy of Chiffon *in trans* in flies carrying two *chiffon* null alleles. Expression of the Chiffon FL transgene fully restored viability and fertility to flies carrying the two *chiffon* null alleles.

### RNA-seq

GFP-positive stage 12–14 embryos were manually selected using a dissecting microscope with fluorescence (Nightsea SFA). Total RNA from six embryos (stage 12–14) per biological replicate was extracted using a Direct-zol RNA microprep kit (R2060, Zymo Research). Four biological replicates were performed for RNA-seq experiments. Libraries were generated from 25 ng RNA using an Ovation RNA-seq system (NuGEN) with unique dual indices for multiplexing and *Drosophila*-specific ribodepletion.

### RNA-seq analysis

Reads were trimmed using Trimmomatic (v0.38). Quality trimmed reads were mapped to the *D. melanogaster* genome (BDGP6.99) using HISAT2 (v2.0). Counts were identified for each gene using Htseq-count (v0.11.1) with default parameters. Counts were normalized by replicates using RUV normalization (R package RUVseq, v1.26.0). DEGs [false discovery rate (FDR)<0.05, FC≥0.5] were identified using EdgeR (v3.30.3) filtering low count samples, removing rRNA genes because RNA-seq libraries were ribodepleted and removing the following features: ‘no feature’, ‘ambiguous’, ‘too low aQual’, ‘not aligned’ and ‘alignment not unique’. GO term analysis was performed with clusterProfiler (v 3.18.1) and TopGO (v2.44.0).

### ChIP

*S. cerevisiae* chromatin was prepared as described previously (Cloutier et al., 2013). GFP-positive stage 12–14 *Drosophila* embryos were manually selected using a dissecting microscope with fluorescence (Nightsea SFA). Three hundred GFP-positive embryos per biological replicate were collected. After dechorionation with 50% bleach, *Drosophila* embryos were fixed as previously described (Zeitlinger et al., 2007). Briefly, embryos (stage 12–14) were transferred to a 3 ml glass vial with PBT (PBS with 0.1% Triton X-100). PBT was then replaced with 230 µl fixation solution (50 mM HEPES, 1 mM EDTA, 0.5 mM EGTA, 100 mM NaCl) plus 1.8% formaldehyde and 750 µl n-heptane. Embryos were shaken vigorously for 15 min at room temperature. They were then centrifuged for 1 min at 500 ***g*** at 4°C, and the supernatant was discarded. The fixation reaction was quenched by addition of 1.5 ml PBT-glycine (PBT with 250 mM glycine), followed by vigorous shaking for 1 min at room temperature and collection by centrifugation as above. Finally, embryos were washed twice with 1 ml PBT and resuspended in 1 ml PBS with 0.5% Triton X-100. Three hundred GFP-positive embryos (per replicate) were manually collected using a dissecting microscope with fluorescence (Nightsea SFA) and snap frozen in liquid nitrogen. Then, the chromatin pellet was washed three times with buffer A1 (15 mM HEPES, pH 7.5, 15 mM NaCl, 60 mM KCl, 4 mM MgCl_2_, 0.5% Triton X-100) and once with buffer A2 (15 mM HEPES, pH 7.5, 140 mM NaCl, 1 mM EDTA, 0.5 mM EGTA, 1% Triton X-100, 0.1% sodium deoxycholate, 0.1% SDS, 0.5% N-laurosylsarcosine), and finally chromatin was sonicated in 130 µl buffer A2 in Covaris E220 with the following conditions: 14 min, 2% duty cycle, 105 W and 200 cycles per burst to obtain an average fragment size of ∼400 bp. After centrifugation at 20,000 ***g*** for 10 min at 4°C, soluble chromatin was diluted with buffer A2 (0.1% SDS) and used for ChIP. ChIP was performed as described (Jauregui-Lozano et al., 2021) with the following modification: 1 µg *Drosophila* chromatin (with 50 ng *Saccharomyces* spike-in chromatin) was used to enable us to normalize our signal to this internal control (Chen et al., 2015). Spike-in factors are reported in Table S3. Chromatin was incubated with 1 µg of each of the following antibodies: anti-acetylated H3-Lys14 (rabbit, 07353, Millipore), anti-H3 (rabbit, ab1791, Abcam) at 4°C overnight with rotation. Immunoprecipitated protein-DNA complexes were incubated with 25 μl Dynabeads protein G (10004D, ThermoFisher Scientific) for 4 h at 4°C. Protein–DNA complexes were eluted from the magnetic beads with Elution buffer (1× TE, 1% SDS, 250 mM NaCl), treated with RNAse A (EN0531, ThermoFisher Scientific) at 37°C for 30 min and with Proteinase K (AM2546, ThermoFisher Scientific) at 55°C overnight. DNA purification, quantification, and input fragment size determination were performed as previously described (Jauregui-Lozano et al., 2021). Three biological replicates were performed for ChIP-seq experiments. ChIP-seq libraries were generated from 1 ng input and 0.5 ng ChIP DNA using an Ovation Ultralow library system (NuGEN) with unique dual indices for multiplexing.

### ChIP-seq analysis

Reads were trimmed using Trimmomatic (v0.38) to filter out low quality reads and remove adapter contamination. Quality trimmed reads were mapped to the *D. melanogaster* genome (BDGP6.99) and *S. cerevisiae* (S288C) genome using Bowtie2 (v2.3.5.1) using -sensitive settings. For H3K14ac signal, spike-in factors were calculated as described (Orlando et al., 2014) (reported in Table S3) and used to generate normalized bigwig files using deepTools (v3.1.1) bamCoverage subpackage, generating reference-adjusted reads per million (RRPM). Metaplots and genomic distribution heatmaps were made with deepTools (v3.1.1) subpackages: computeMatrix, plotHeatmap and plotProfile. The Integrative Genomics Viewer (IGV) was used to generate single gene examples shown.

### Immunostaining

Embryos were dechorionated with bleach and crosslinked with 4% formaldehyde in PEM buffer (0.5 M PIPES, 5 mM MgCl_2_, 5 mM EGTA, pH 6.9) in 2 ml heptane, while vortexing at medium speed for 20 min. Embryos were devitellinized in methanol/heptane and kept at −20C until needed. Embryos were immunostained as in Rothwell and Sullivan (2007) using anti-H3K14ac (1:200; rabbit, 07353, Millipore), anti-HA (1:200; rat, 11867423001, Roche), anti-FLAG (1:200; rabbit, F7425, Sigma-Aldrich), anti-*Drosophila* Chiffon-C (1:200; rabbit), Alexa Fluor 488- and Alexa Fluor 568- conjugated secondary antibody (1:400; goat, Thermo Fisher Scientific). Cell nuclei were stained using 1 µg/ml DAPI. Embryos were staged according to the number of nuclei stained by DAPI. Images were taken using a Leica DM6B fluorescent microscope equipped with CTR6-LED and DFC450 digital camera. Acetylation levels were determined as average sum intensity values for fluorescence comparing GFP (mutant embryo) with the non-GFP embryo (WT homozygous sibling) using ImageJ software. Multiple sections were examined for each embryo (*n*≥3), and single optical sections are shown for each representative image.

### Cloning and purification of recombinant Gcn5 core complex from Sf21 insect cells

Coding sequences for Ada2b-PA, Gcn5, Ada3 and Sgf29 were cloned into pBACPAK8 vectors with the addition of an N-terminal His-FLAG epitope-tag for Ada2b-PA, and expressed in Sf21 cells infected with Baculovirus as previously described (Stephenson et al., 2015).

### Western blotting

The following antibodies were used for western blot analysis: anti-Gcn5 (1:1000; rabbit), anti-Ada3 (1:3000; rabbit), anti-Sgf29 (1:500; rabbit), anti-FLAG M2-peroxidase (HRP) (1:5000; A8592, Sigma-Aldrich).

### qRT-PCR

qRT-PCR analysis for mRNA levels of Chiffon-A or Chiffon-B during the early developmental stage was performed on RNA isolated from single embryos collected 0–3 h AEL using a Direct-zol RNA Micro-prep kit (R2062, Zymo Research). Relative expression for each gene was normalized to *RpL32*. Primers are listed in Table S5.

### Germline clone analysis

*hsFLP/Y; ovoD1, FRT40A/Cy* males were crossed to females of the indicated genotype, e.g. *chiffon^ETBE3^, FRT40A/CyO* that were homozygous for rescue transgene on chromosome 3 (see Table S3 for genotypes). Progeny were heat shocked for 2 h on two subsequent days 3–4 days AEL, and non-CyO females were selected and crossed with WT (*w^1118^*) males to assess fertility and embryo hatch rates. Non-production of eggs, hatched and non-hatched embryos were counted for individual female progeny (*n*). Unhatched embryos were defined as failure to produce first-instar larvae >26 h AEL.

### Antibody production

The Chiffon-C polyclonal antibody was generated against His-tagged 1400–1695 aa C-terminal recombinant Chiffon protein expressed in *Escherichia coli* injected into rabbits. The rabbit serum was affinity purified against GST-tagged recombinant Chiffon C-terminal domain and used for immunostaining as described.

## Supplementary Material

Supplementary information

Reviewer comments
