# Supplementary material for: Chiffon triggers global histone H3 acetylation and expression of developmental genes in Drosophila embryos
Source: J Cell Sci. 2022 Jan 24;135(2):jcs259132. doi: 10.1242/jcs.259132 (PMC8917357; doi:10.1242/jcs.259132)
Supplement: Supplementary information [file joces-135-259132-s1.pdf]

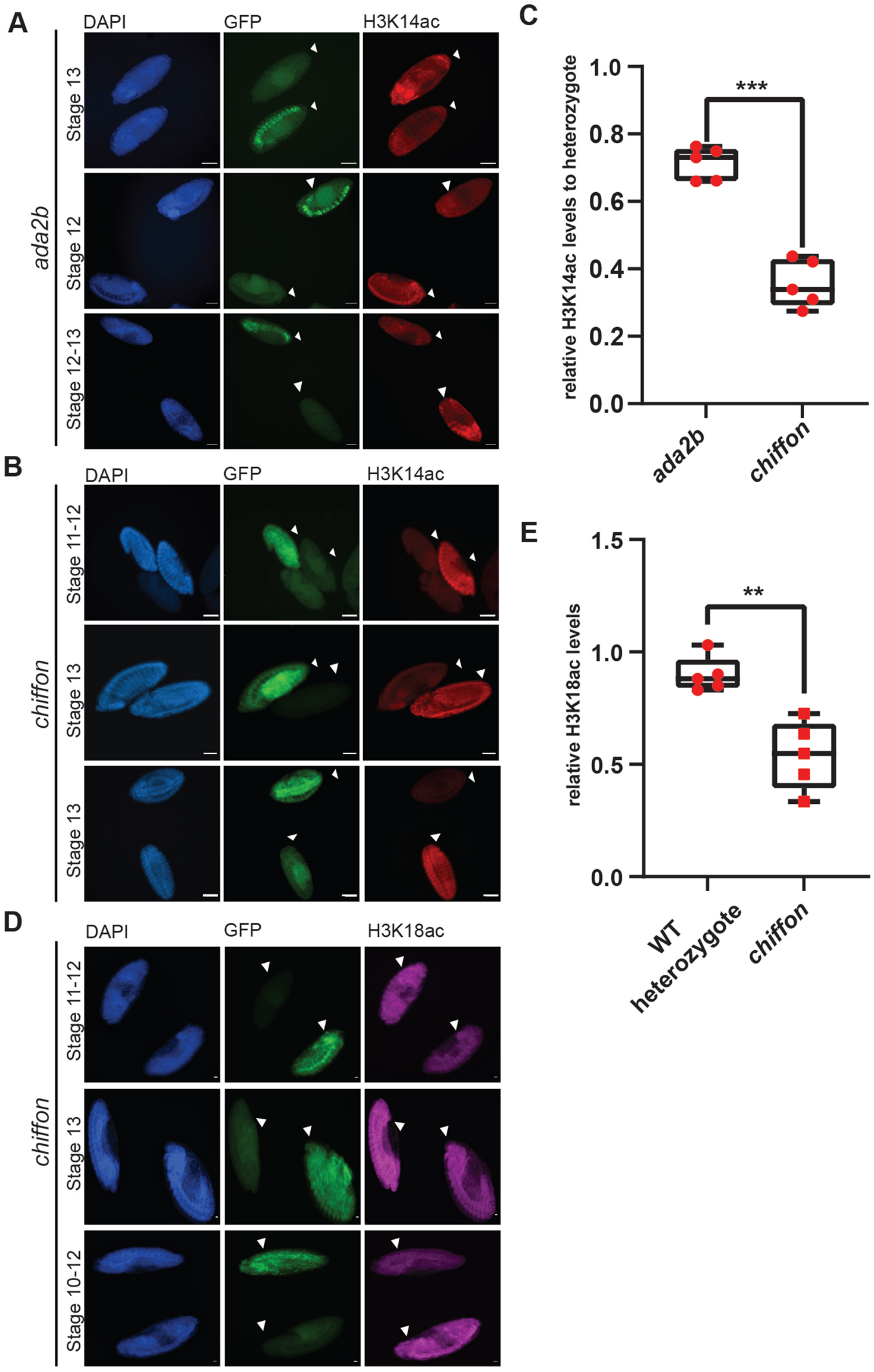

Fig. S1. Chiffon is required for H3K14ac in embryos to a greater extent than Ada2b. (A) Stage 10 – 13 *ada2b* embryos were examined for DAPI and H3K14ac. GFP-positive embryos are *ada2b* null. Scale bars: 20 µm. (B) Stage 11 - 13 *chiffon* embryos were examined for DAPI and H3K14ac. GFP-positive embryos are *chiffon* null. Scale bars: 20 µm. (C) Box plots showing relative H3K14ac levels in GFP versus non-GFP (heterozygote siblings) embryos. 5 independent embryos were quantified. *p*-value (***, $p < 0.0001$) for the indicated comparison was determined by t-test. (D) Stage 10 – 13 *chiffon* embryos were examined for DAPI and H3K18ac. GFP-positive embryos are *chiffon* null. Scale bars: 20 µm. (E) Box plots showing relative H3K18ac levels in GFP versus non-GFP (heterozygote siblings) embryos. 5 independent embryos were quantified. *p*-value (**, $p < 0.0016$) for the indicated comparison was determined by t-test.

Journal of Cell Science • Supplementary information

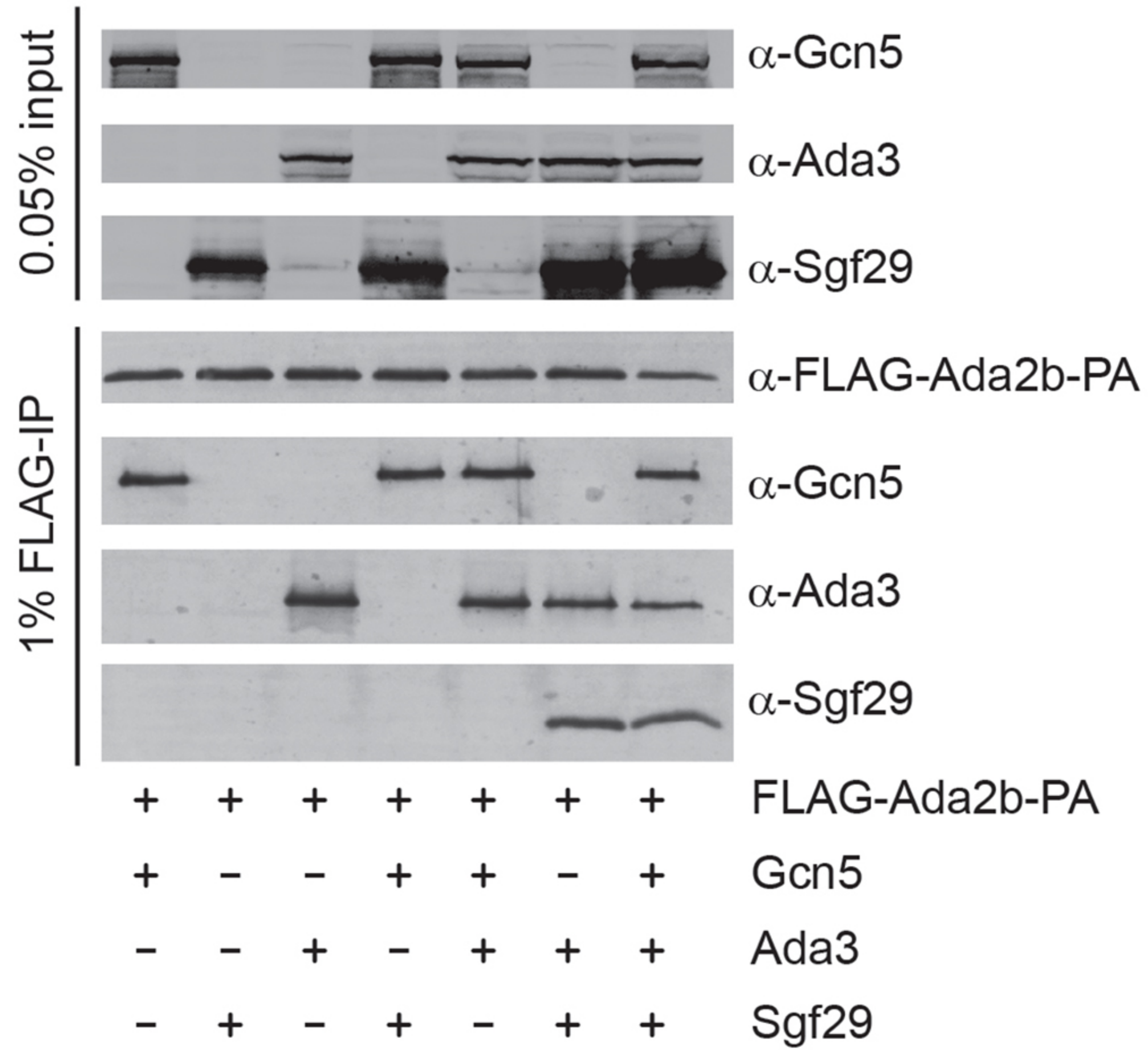

Fig. S2. Ada2b-PA is necessary for formation of the Gcn5 core HAT module. (A) Gcn5, Ada3, Sgf29, and Ada2b-PA (N-terminal FLAG tag) were expressed in Baculovirus-infected Sf21 cells, and cell lysates were mixed in the indicated pairwise combinations, and subjected to anti-FLAG affinity chromatography. The resulting immunopurified complexes were eluted, and assess for presence of the indicated proteins by western blotting analysis.

Journal of Cell Science • Supplementary information

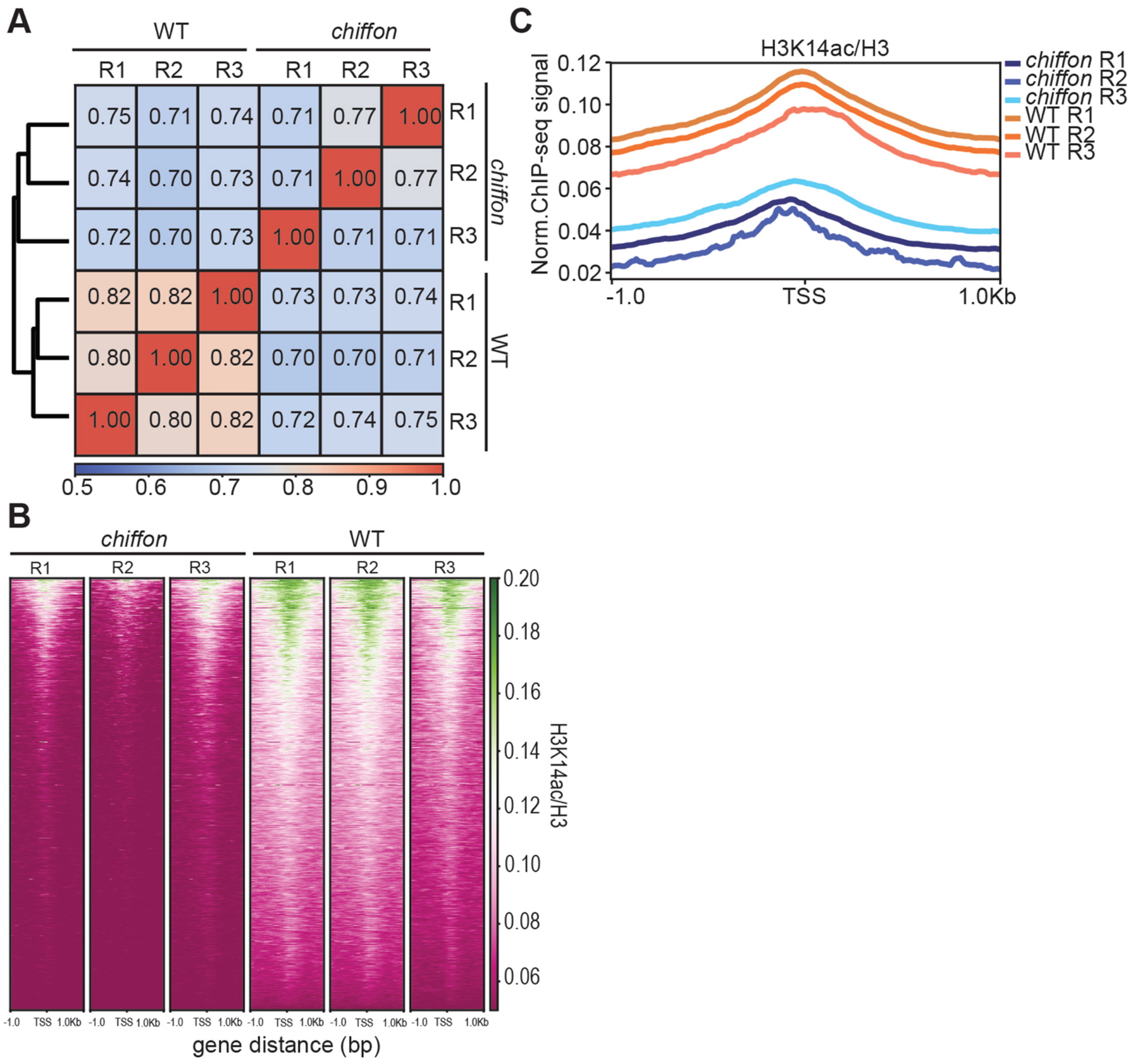

Fig. S3. CHAT is required for global H3K14ac levels in embryos. (A) Spearman correlation heatmap of H3K14ac ChIP-seq data comparing *chiffon* and WT (*chiffon* + FL) samples. (B) Heatmaps showing RRPM-normalized H3K14ac ChIP-seq signal around the TSS of protein-coding genes comparing *chiffon* and WT. (C) Metaplot of RRPM-normalized H3K14ac ChIP-seq signal around the transcription start site (TSS) averaged for all protein-coding genes in *chiffon* and WT samples for each replicate.

Journal of Cell Science • Supplementary information

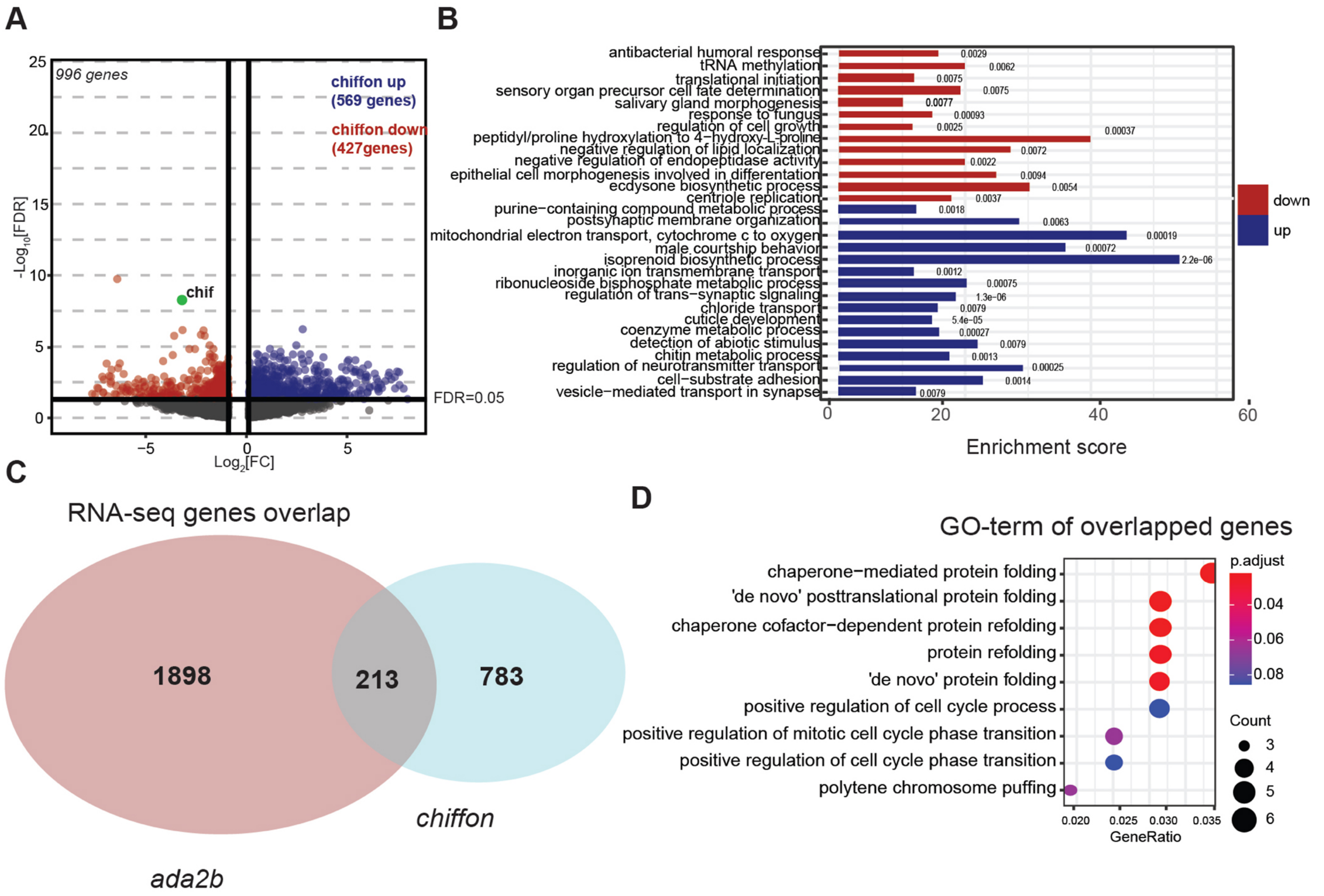

Fig. S4. Chiffon is necessary for expression of developmental genes. (A) Volcano plot showing the fold change of differential expressed genes of CHAT regulated genes plotted as log2(fold change in counts per million reads, CPM) for each gene relative to its p-value (−log10[FDR]). The dashed line shows the statistical significance cut-off (FDR<0.05) used for DEG analysis. (B) Over-represented GO terms ($p < 0.01$, Fisher's exact test) were identified for 427 downregulated or 569 upregulated genes relative to all 9765 expressed genes using TopGO. (C) Venn diagram indicating the overlapping genes detected in the *chiffon* and *ada2b* RNA-seq analysis. (D) GO terms analysis on *chiffon* and *ada2b* commonly regulated genes.

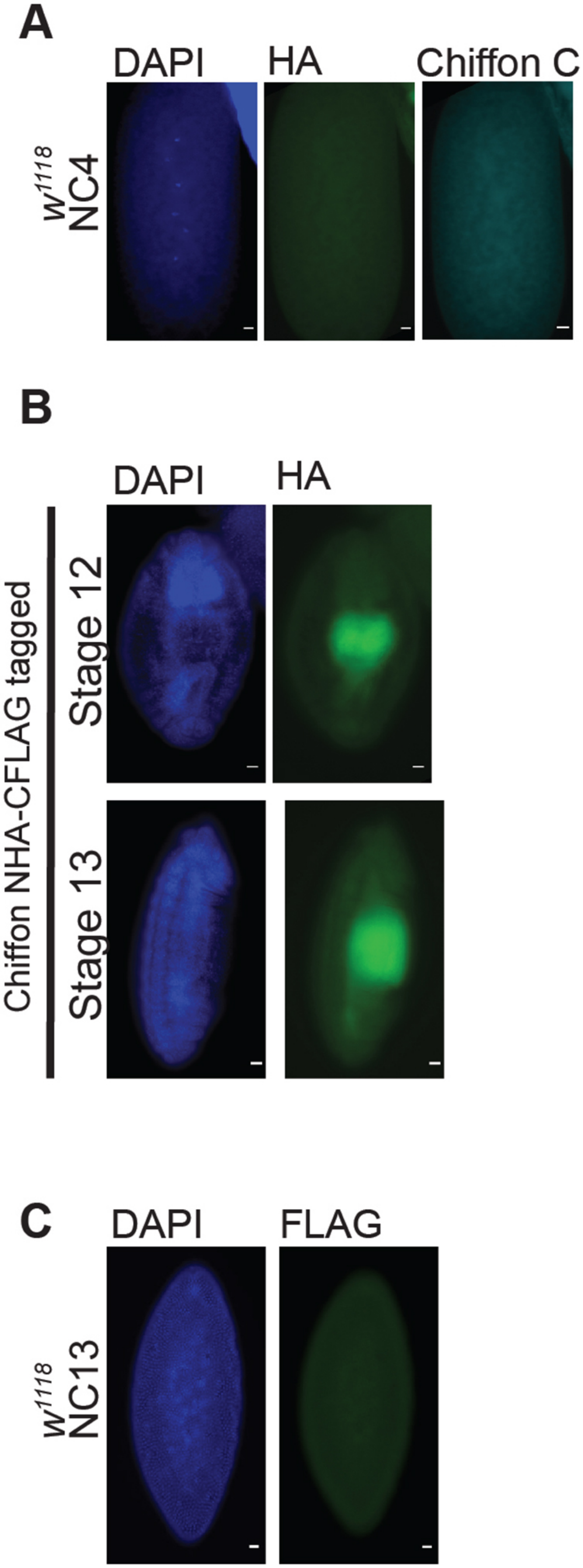

Journal of Cell Science • Supplementary information

Fig. S5. Specificity of the HA and FLAG antibodies for embryo immunostaining. (A) Untagged wild-type embryos (*w^{1118}*) at NC4 immunostained for DAPI, HA, and Chiffon C-terminal antibody showing lack of background signal under the conditions used, and no expression of Chiffon-B in early embryos. Identical immunostaining and imaging conditions were used for all other HA immunostaining experiments. (B) Epitope-tagged Chiffon-FL stage 12 and 13 embryos were examined for DAPI and HA. Scale bars: 20 µm. The green signal represents auto-fluorescence from the gut tissue because we did not detect any HA (Chiffon-A) signal in this developmental stage. (C) Untagged wild-type embryo (*w^{1118}*) at NC13 immunostained for DAPI and FLAG showing lack of background signal under the conditions used.

Table S1. Summary of DEGs in ada2b RNA-seq.

Click here to download Table S1

Table S2. Summary of DEGs in chiffon RNA-seq.

Click here to download Table S2

SUPPLEMENTAL REFERENCES FOR TABLE S2

Orlando DA, Chen MW, Brown VE, Solanki S, Choi YJ, Olson ER, Fritz CC, Bradner JE, Guenther MG. 2014. Quantitative ChIP-Seq normalization reveals global modulation of the epigenome. *Cell Rep* **9**: 1163–1170.

Stephenson R, Hosler MR, Gavande NS, Ghosh AK, Weake VM. 2015. Characterization of a Drosophila Ortholog of the Cdc7 Kinase: A ROLE FOR Cdc7 IN ENDOREPLICATION INDEPENDENT OF CHIFFON*. *Journal of Biological Chemistry* **290**: 1332–1347.

Torres-Zelada EF, Stephenson RE, Alpsoy A, Anderson BD, Swanson SK, Florens L, Dykhuizen EC, Washburn MP, Weake VM. 2019. The Drosophila Dbf4 ortholog Chiffon forms a complex with Gcn5 that is necessary for histone acetylation and viability. *J Cell Sci* **132**: jcs214072.

Weake VM, Dyer JO, Seidel C, Box A, Swanson SK, Peak A, Florens L, Washburn MP, Abmayr SM, Workman JL. 2011. Post-transcription initiation function of the ubiquitous SAGA complex in tissue-specific gene activation. *Genes Dev* **25**: 1499–1509.

Table S3. Spike-in factors for ChIP-seq analysis. Spike-in factors for each ChIP-seq sample were calculated following the method described in (Orlando et al. 2014).

Click here to download Table S3

Journal of Cell Science • Supplementary information

Table S4. Summary of *Drosophila* stocks used in this study. Genotypes and sources of stocks including original description of any transgenes (ada2b, chiffon) are indicated.

Fly Stock	Description	Genotype	Source/Reference
*W^1118^*	Control untagged stock for immunostaining and germline clones	*W^1118^*	BDSC 3605
chiffon^DsRed^, actin-Gal4	*chiffon* null allele on chromosome 2 with actin-Gal4 for GFP sorting of *chiffon* mutant embryos	*P{w[+mC]=Act5C-GAL4}25FO1, chif^[DsRed-attP]^/CyO*	This study
chiffon^DsRed^, actin-Gal4; chiffonFL	*chiffon* null allele on chromosome 2 with actin-Gal4 plus a transgene expressing full length (1 – 1695aa) for GFP sorting.	*yw; P{w[+mC]=Act5C-GAL4}25FO1, chif^[DsRed-attP]^/CyO; P{w+mC =ChiffonFL]-NHACFLAG_ chifp}attP2*	This study; Chiffon transgenes described in (Torres-Zelada et al. 2019)
chiffon^DsRed^, actin-Gal4; chiffonFL^WF24^	*chiffon* null allele on chromosome 2 with actin-Gal4 plus a transgene expressing FL^WF24^ (1 – 1695aa) containing a nonsense mutation at 174aa (c.520C>T; p.174Q>X) for GFP sorting.	*yw; P{w[+mC]=Act5C-GAL4}25FO1, chif^[DsRed-attP]^/CyO; P{w+mC =Chiffon FL^WF24^4-NHACFLAG_ chifp}attP2*	This study; Chiffon transgenes described in (Torres-Zelada et al. 2019)
chiffon^DsRed^, actin-Gal4; chiffonΔN	*chiffon* null allele on chromosome 2 as actin-Gal4 plus a transgene expressing ΔN (401 – 1695aa) for GFP sorting.	*yw; P{w[+mC]=Act5C-GAL4}25FO1, chif^[DsRed-attP]^/CyO; P{w+mC =ChiffonΔN-NHACFLAG_ chifp}attP2*	This study; Chiffon transgenes described in (Torres-Zelada et al. 2019)
chif^ETBE3^, 10XUAS:GFP	*chiffon* null allele on chromosome 2 with 10XUAS:GFP for GFP sorting of *chiffon* null mutants embryos	*chif^[ETBE3]^, P{w[+mC]=10XUAS-IVS-mCD8::GFP}attP40*	This study
10XUAS-IVS-mCD8:GFP	GFP control stock for GFP sorting of *chiffon* mutants	*w^*^; P{y[+t7.7] w[+mC]=10XUAS-IVS-mCD8::GFP}attP40*	BDSC 32186
Act5C-GAL4	Actin-Gal4 control stock for GFP sorting of *chiffon* mutants	*y^1^ w[*]; P{w[+mC]=Act5C-GAL4}25FO1/CyO, y[+]*	BDSC 4414
ada2b^1^, 10XUAS:GFP	*ada2b* null allele on chromosome 3 with 10XUAS:GFP for GFP sorting of *ada2b* mutant embryos	*ada2b^[1]^, P{w[+mC]=10XUAS-IVS-mCD8::GFP}su(Hw)attP1/TM3, Sb^[1]^*	This study
ada2b^842^, elav-GAL4	*ada2b* null allele on chromosome 3 with elav-GAL4 for GFP sorting of *ada2b* mutant embryos	*ada2b^[842]^, w[*]; P{w[+mC]=elav-GAL4}LL7/TM3, Sb^[1]^*	This study
*ada2b^1^*, 10XUAS:GFP; ada2b-PA	*ada2b* null allele on chromosome 3 with 10XUAS:GFP plus a transgene expressing Ada2b-PA isoform	*yw; P{w+mC =ADA2B-PA_ ada2bEN}attP40; ada2b^[1]^, w^+mC^=10XUAS-IVS-mCD8::GFP}su(Hw)attP1*	This study; Ada2b transgenes described in (Weake et al. 2011)
*ada2b^1^*, 10XUAS:GFP; ada2b-PB	*ada2b* null allele on chromosome 3 with 10XUAS:GFP plus a transgene expressing Ada2b-PB isoform	*yw; P{w+mC =ADA2B-PB_ ada2bEN}attP40; ada2b[1], w[+mC]=10XUAS-IVS-mCD8::GFP}su(Hw)attP1ada2b^[1]^*	This study; Ada2b transgenes described in (Weake et al. 2011)

Journal of Cell Science • Supplementary information

ada2b[842], elav4-GAL4; ada2b-PB	*Ada2b* null allele on chromosome 3 with elav-GAL4 plus a transgene expressing Ada2b-PA isoform	*yw; P{w+mC =ADA2B-PB_ ada2bEN}attP40; ada2b[842], w[*]; P{w[+mC]=tubP-GAL4}LL7/TM3, Sb[1]*	This study; Ada2b transgenes described in (Weake et al. 2011)
10XUAS-IVS-mCD8::GFP	GFP control stock for GFP sorting of ada2b mutants	*w[1118]; P{y[+t7.7] w[+mC]=10XUAS-IVS-mCD8::GFP}su(Hw)attP1*	BDSC 32187
Elav4-GAL4	GFP control stock for GFP sorting of ada2b mutants	*w[1118]; P{y[+t7.7] w[+mC]=GMR27E06-GAL4}attP2*	BDSC 45530
Ada2b-PB-HF2	Ada2b-PB HA and FLAG tagged in the C-terminal for immunostaining	*yw; P{w+mC =ADA2B-PB-HF2_ada2bEN}attP40*	This study
Ada2b-PA-HF2	Ada2b-PB HA and FLAG tagged in the C-terminal for immunostaining	*yw; P{w+mC =ADA2B-PA-HF2_ada2bEN}attP40*	This study
chiffon[ETBE3]*/chiffon*[DsRed]*;* ChiffonFL-NHACFLAG	Chiffon FL HA tagged in the N-terminal and FLAG tagged in the DN-terminal rescue on *chiffon* null mutant background	*yw; chif[ETBE3], P{ry[+t7.2]=neoFRT}40A/ chiffon[DsRed-attP]; P{w+mC =ChiffonFL-NHACFLAG_ chifp}attP2*	This study
chiffon [ETBE3], FRT40A	*chiffon, FRT40A* stock for germline clone experiment	*chif[ETBE3], P{ry[+t7.2]=neoFRT}40A/CyO*	(Stephenson et al. 2015)
chiffon [ETBE3], FRT40A; chiffonΔN (FLAG-HA)	*chiffon, FRT40A* stock plus transgene expressing ΔN (401 – 1695aa) for germline clone experiment	*yw; chif[ETBE3], P{ry[+t7.2]=neoFRT}40A/CyO; P{w+mC =ChiffonDN-NHACFLAG_ chifp}attP2*	This study
chiffon [ETBE3], FRT40A; chiffonN[376S>X] (FLAG-HA)	*chiffon, FRT40A* stock plus transgene expressing N (1 – 400aa) for germline clone experiment	*yw; chif[ETBE3], P{ry[+t7.2]=neoFRT}40A/CyO; P{w+mC =ChiffonN[376S>X]-NHACFLAG_ chifp}attP2/MKRS*	This study
chiffon [ETBE3], FRT40A; chiffonFL (FLAG-HA))	*chiffon, FRT40A* stock plus a transgene expressing full length (1 – 1695aa) for germline clone experiment	*yw; chif[ETBE3], P{ry[+t7.2]=neoFRT}40A/CyO; P{w+mC =ChiffonFL-NHACFLAG_ chifp}attP2/MKRS*	This study
FRT40A, ubi-nlsGFP	FRT40A control stock for germline clone experiment	*w[1118]; P{w[+mC]=Ubi-GFP(S65T)nls}2L P{ry[+t7.2]=neoFRT}40A/CyO*	BDSC 5629
hsFLP; Adv[1]*/CyO*	Heat-shock FLP, Adv stock for germline clone experiment	*P{ry[+t7.2]=hsFLP}1, w[1118]; Adv[1]/CyO*	BDSC 6
ovoD1, FRT40A	ovoD1 FRT40 stock used for germline clone analysis	*P{w[+mC]=ovoD1-18}2La, P{ry[+t7.2]=neoFRT}40A/Dp(?;2)bw[D], S[1] wg[Sp-1] Ms(2)M[1] bw[D]/CyO*	BDSC 2121

Journal of Cell Science • Supplementary information

Table S5. Primer sequences used in this study. Primers used for qRT-PCR analysis in single embryos. Forward and Reverse primer sequences per each set of primer are indicated.

		**Sequence**
Rpl32	Forward	5'- CTTCTGGTTTCCGGCAAGGTA-3'
	Reverse	5'- GGTCGAAATACGAGCCGCA-3'
Nanos	Forward	5'-ACTTTGAGTCCGCCCATTAC-3'
	Reverse	5'-GTGGTACTGTCGCTGCATAA-3'
even skipped	Forward	5'-CCTGGTTGTGGACCTCTTG-3'
	Reverse	5'-TGCCGTTCAAGGAGTTATCC-3'
HA-*chiffon* *(Chiffon-A)*	Forward	5'-TCCTTACGACGTACCAGACTAT-3'
	Reverse	5'-CTGCTGTTGAGTGGCTAGTT-3'
FLAG-*chiffon* *(Chiffon-B)*	Forward	5'-ATCTCGCAGTTCCTGAAGAAG-3'
	Reverse	5'-TGTCATCATCGTCCTTGTAGTC-3'

Journal of Cell Science • Supplementary information
