# Supplementary material for: Chiffon triggers global histone H3 acetylation and expression of developmental genes in Drosophila embryos
Source: J Cell Sci. 2022 Jan 24;135(2):jcs259132. doi: 10.1242/jcs.259132 (PMC8917357; doi:10.1242/jcs.259132)
Supplement: Reviewer comments [file joces-135-259132_review_history.pdf]

Chiffon triggers global histone H3 acetylation and expression of developmental genes in *Drosophila* embryos

Eliana F. Torres-Zelada, Smitha George, Hannah R. Blum and Vikki M.Weake
DOI: 10.1242/jcs.259132

Editor: David Glover

Review timeline
Original submission: 13 July 2021
Editorial decision: 3 September 2021
First revision received: 5 October 2021
Editorial decision: 16 November 2021
Second revision received: 24 November 2021
Accepted: 6 December 2021

Original submission

First decision letter

MS ID#: JOCES/2021/259132

MS TITLE: CHAT acts upstream of other Gcn5 complexes during early Drosophila embryogenesis, triggering global histone H3 acetylation and expression of developmental genes

AUTHORS: Eliana F. Torres-Zelada, Smitha George, Hannah R. Blum, and Vikki Marie Weake
ARTICLE TYPE: Research Article

We have now reached a decision on the above manuscript.

To see the reviewers' reports and a copy of this decision letter, please go to: https://submit-jcs.biologists.org and click on the 'Manuscripts with Decisions' queue in the Author Area. (Corresponding author only has access to reviews.)

As you will see, the reviewers raise a number of substantial criticisms that prevent me from accepting the paper at this stage. They suggest, however, that a revised version might prove acceptable, if you can address their concerns. If you think that you can deal satisfactorily with the criticisms on revision, I would be pleased to see a revised manuscript. We would then return it to the reviewers.

We are aware that you may be experiencing disruption to the normal running of your lab that makes experimental revisions challenging. If it would be helpful, we encourage you to contact us to discuss your revision in greater detail. Please send us a point-by-point response indicating where you are able to address concerns raised (either experimentally or by changes to the text) and where you will not be able to do so within the normal timeframe of a revision. We will then provide further guidance. Please also note that we are happy to extend revision timeframes as necessary.

Please ensure that you clearly highlight all changes made in the revised manuscript. Please avoid using 'Tracked changes' in Word files as these are lost in PDF conversion.

© 2021. Published by The Company of Biologists under the terms of the Creative Commons Attribution License (https://creativecommons.org/licenses/by/4.0/).

I should be grateful if you would also provide a point-by-point response detailing how you have dealt with the points raised by the reviewers in the 'Response to Reviewers' box. Please attend to all of the reviewers' comments. If you do not agree with any of their criticisms or suggestions please explain clearly why this is so.

Reviewer 1

Advance summary and potential significance to field

Provides the first indication of how distinct GCN5-containing histone acetyltransferase complexes, SAGA/ADA vs CHAT, influence gene expression programs important to gene expression in flies. The studies also reveal for the first time that the CHAT complex also impacts histone acetylation levels independently of Gcn5.

Comments for the author

The Gcn5 histone acetyltransferase is important gene regulation in organisms ranging from yeast to mammals as part of the SAGA complex. However, Gcn5 also functions within other complexes, and understanding the “division of labor” between these complexes is a major question in the field. Torres-Zelada et al address this question in flies, where Gcn5 is part of distinct ATAC, SAGA/ADA and CHAT complexes. These complexes incorporate different Ada2 subunits, with ATAC housing Ada2A and SAGA/ADA and CHAT housing different splice variants of Ada2b. The authors make use of these differences to genetically inactivate SAGA/ADA or CHAT in fly embryos, and then comparing changes in gene expression histone acetylation and development. They also mutate the chiffon gene, which encodes two gene products, Db4 which regulates Cdc7, and Chiffon-B, which interacts with Gcn5 to nucleate formation of the CHAT complex.
The authors find that almost all 2000+ genes regulated by SAGA/ADA are also regulated CHAT in fly embryos. They also identified 500+ genes that are uniquely regulated by CHAT. Surprisingly, they found that chiffon mutations that selectively disrupt CHAT functions have an even greater effects on histone H3 acetylation than do mutations in ada2b. These data suggest that chiffon/CHAT may influence the activity or targeting of multiple KATs beyond Gcn5.
Overall, the studies presented here are well done, using a systematic approach which judicious interpretation of data. The mechanisms by which CHAT vs SAGA are targeted (or not) to certain genes is not yet clear. Nor is it clear how CHAT influences global levels of H3 acetylation. Nonetheless, these studies constitute a significant step forward in understanding Gcn5 and CHAT functions.
Minor suggestion: early on, provide the reader with a small diagram indicating the differences in CHAT vs. SAGA/ADA composition.

Reviewer 2

Advance summary and potential significance to field

In the manuscript by Torres-Zelada et al., the authors provide evidence that histone acetyltransferase (HAT) complexes, specifically, the Chiffon histone acetyltransferase (CHAT) complex, is critical for gene expression in the Drosophila early embryo. Drosophila contains four Gcn5-containing complexes (SAGA, ATAC, CHAT and ADA) but it has been challenging to discern whether these HAT complexes support overlapping of distinct gene expression and/or have additional roles beyond regulating gene expression. Gcn5-binding protein Ada2b has two splice isoforms that are differentially acting; Ada2b-PB isoform is in SAGA; in contrast, Ada2b-PA associates with Gcn5, Ada3, Sgf29 and Chiffon forming the CHAT complex. Chiffon is the Drosophila ortholog of Dbf4, which binds and activates the cell cycle kinase Cdc7 to initiate DNA replication. Therefore, by comparing/contrasting Ada2b-PA and Chiffon mutant phenotypes, the authors set out to determine the full function of the CHAT complex.

Through this analysis, the authors conclude that a temporal switch in Chiffon function - supporting DDK/Cdc7 function prior to nc10/11 and then switching to support of CHAT subsequently. This model is supported by RNA-seq analysis that shows most of the Chiffon mutant gene expression

© 2021. Published by The Company of Biologists under the terms of the Creative Commons Attribution License (https://creativecommons.org/licenses/by/4.0/).

changes are due to its support of HAT and not due to its connection to DDK; as well as the finding that the Chiffon-A product that binds Cdc7 is expressed early (NC3-NC14) as for Cdc7. The authors conclude that there is a switch at nc10/11 between the expression of Chiffon-A product that nucleates DDK formation and the Chiffon-B product that nucleates CHAT formation.

The authors' model is attractive and timely; however, the study is not yet convincing. Staging is questionable (e.g. Fig. 4B,D) including using circular arguments about phenotypes to define stage (Fig. S4B); normalization methods are not clearly described (e.g. Fig. 2G); and insufficient information provided in the methods/figure legends that make understanding experiments very challenging (e.g. Fig. 2D stage of embryos?). Major issues are present (see below) that need to be addressed before the paper is ready for publication.

Comments for the author

Major concerns:

1. Experimental design and methods: In general, much more attention must be made to the description of experiments including attention to stage of embryos sample size, as well as to quantitative and statistical analyses.
This is a problem throughout the text but here are some representative examples of how important information was omitted:
i) The embryo collection timepoint used for the RNA-seq experiments is unstated;
it is unclear what stage embryos are represented and how much variation would be expected.
ii) The method that was used for in situ quantification is not described withi(e.g. Fig. S1C - nor was it included in figure legend).
iii) In Fig S3A, the authors say that they observed significantly higher expression of the ΔN transgene, however, no statistical data is provided.
iv) In Fig. 3C, the authors say that 26 genes were differentially expressed between the 2 genotypes that they are describing, however, the shown data suggest that the number of genes might be smaller.
v) At line 239, it is stated that Chiffon-A expression is observed as early as NC3. How were embryos staged? Identifying embryos from nc3-nc8 can be very challenging because nuclei have not all migrated to the periphery. In addition the authors should costain Chiffon NHA-CFLAG with Cdc7.
vi) Lastly, many embryo images need to be replaced with healthy looking embryos in order to be able to make safe conclusions about gene expression. For example in Fig 4D, nc6/7 DAPI staining of Ada2b-PB-FLAG tagged sample, the embryo looks unhealthy/dead.

2. Embryos staging: The lack of H3K14ac staining of embryos at stage 13 (figure 1B) is convincing. On the other hand, gene expression in the early embryo is very dynamic and the embryo stage of samples is unclear (e.g. Fig. 1C-E). More careful description of the staging is required; it is hard to evaluate the study properly without this information.
Furthermore, as mentioned above, the embryos in Figure 4B and D need to be replaced with healthy, stage appropriate ones.
The stage of the embryos used in each case should be clearly stated either in the main text or in each figure's caption.

3. Additional references: The authors should place their study in context of the current field. Several studies have examined the role of other ubiquitously expressed timing factors. Are there any timing factors that might relate specifically, to CHAT action proposed by the authors to initiate at nc10/11?
4. Data interpretation: In Fig. S2D, the authors argue for global decrease in histone acetylation (not only at CHAT-regulated genes). How can specific targets of CHAT then be identified?
5. The GEO data should be available to the reviewers for evaluation during the review process. Please provide the reviewers' with a token for access.
5. Additional support for the model that Chiffon supports entry of Ada2b-PB into the nucleus. In the DN Chiffon mutant, does HA-Ada2-PA enter the nucleus at an earlier stage?

Minor comments:
What is known about the ability of Chiffon to regulate HeK9ac levels? If this has been investigated please add to the intro and/or discussion.

© 2021. Published by The Company of Biologists under the terms of the Creative Commons Attribution License (https://creativecommons.org/licenses/by/4.0/).

Line 265: Typo “nucleusnuclei” should be fixed.
Line 276: As stated by the authors, CHAT formation occurs just prior to the de novo large-scale recruitment of RNA pol II that leads to activation of the zygotic genome. It would be helpful to relate CHAT function to that of pioneer factors, Zelda, Odd paired, etc. Could HAT complexes relate to differential action of the multiple pioneer factors acting in the early embryo?
Line 288: The supporting literature for the 2 waves of transcriptional activation should be cited in the paper.
Figure 2G. The scale of the Y axis is missing as well as information regarding the browser that was used to support this analysis.
Figure 4D. Panel Ada2b-PB-FLAG DAPI: the embryo is unhealthy (potentially dead).
Figure S4B. The authors should use standard methods to support embryo staging and present their data accordingly. This could include costaining with genes that initiate expression at particular stages and/or using a quantitative approach to calculate nuclei per area and extrapolate to nc stage.
DDK should be explained in the context of the abstract. To a non-expert, its significance is unclear.

Reviewer 3

Advance summary and potential significance to field

Torres-Zelada et al have compared Ada2b mutants to chiffon mutants in Drosophila with respect to gene expression changes (RNA-seq) and histone acetylation (H3K14ac ChIP-seq). Ada2b mutants disrupt three histone acetyltransferase (HAT) complexes in flies, SAGA, ADA and CHAT. Using rescue transgenes expressing either the PA or PB splice isoforms, the authors identify redundant, cooperative and genes unique to SAGA/ADA or CHAT. Although SAGA/ADA/CHAT are redundant for expression of most genes that are deregulated in Ada2b mutants, CHAT has a stronger effect and controls more uniquely regulated genes.
Surprisingly, chiffon mutants that disrupt CHAT but not SAGA/ADA has a stronger effect on H3K14ac than Ada2b mutants that inactive all three complexes, and the overlap in gene expression changes between chiffon and Ada2b mutants is poor. The chiffon locus is complicated, since it may be regulated translationally.
The authors nicely demonstrate that the chiffon N-terminus that interacts with Cdc7 is required during the early embryonic nuclear cell cycles, whereas the C-terminus that forms CHAT takes over at later embryonic stages.

This is important work that uses genetic analyses to decipher the function of HAT complexes in vivo, but the results are not that easy to interpret. The use of different alleles in trans, the many replicates for RNA-seq and spike-in normalized ChIP-seq ensures high quality data, but the immunostainings and imaging needs to be improved. At present, the conclusions from these stainings are not supported by the images.

Comments for the author

1. The paper would benefit from a more clear description of the experimental setup. It took me some time to realize that the Ada2b rescue transgenes were driven by endogenous promoters and not by UAS sites. Fig. 1A could illustrate this in a better way.
2. I could not find information on what embryonic stage was used for RNA-seq in Ada2b mutants.
3. The quality of the immunostainings and the resulting images need improvement. H3K14ac has previously been shown to be strongest in the CNS, but this is not clear in Fig. 1B and Fig. S1 (Fig. 2B looks better).
4. Figure 4 is even more difficult to interpret. Is the signal from Chiffon-C Ab in NC4 background fluorescence? Did the authors test the specificity of this Ab on chiffon mutants?
5. What is signal and what is background using the HA and FLAG Abs in Fig. 4B and D? The HA signal in Fig. S4A looks like gut autofluorescence.
6. The imaging parameters should be described in the Methods.
7. The stronger effect of chiffon than of Ada2b mutants on H3K14ac is perplexing. Since the authors use two alleles in trans and have previously shown that H3K14ac levels in chiffon mutants

© 2021. Published by The Company of Biologists under the terms of the Creative Commons Attribution License (https://creativecommons.org/licenses/by/4.0/).

can be rescued by chiffon transgenes, this is most likely not caused by second site mutations. The authors should expand their discussion on what could be causing this phenotype.

8. What could be the explanation of the poor overlap of gene expression changes in chiffon and Adab2 mutants? Is there anything from the previous mass spectrometry experiments performed by the authors that indicate a function for Chiffon outside the CHAT and DDK complexes?
9. Given that Chiffon could have other functions not related to CHAT or DDK, the title of the paper should be changed.
10. Figures 2E-G are not mentioned in the text.

First revision

Author response to reviewers' comments

RESPONSE TO REVIEWERS:
We would like to thank the reviewers for their comments and suggestions. We have addressed each comment with our specific responses below (italics). We have made changes to the manuscript text as suggested by each Reviewer, and these changes are highlighted in yellow on the marked manuscript (text). We have made major changes to the text as requested by the editor and reviewers. These changes include a revised title, revised abstract, the addition of a new discussion section, movement of all methods into the main text, and clarifying several points within the results or figure legends as requested. We have also rearranged the figures substantially to address the comments raised, and included two new main figures and one additional supplemental figure. We have also included lists of genes in new supplemental Tables 1 and 2. We provide the page and line numbers for each of these changes in the text under the response to each comment below.

Reviewer #1:

Reviewer #1: The Gcn5 histone acetyltransferase is important gene regulation in organisms ranging from yeast to mammals as part of the SAGA complex. However, Gcn5 also functions within other complexes, and understanding the “division of labor” between these complexes is a major question in the field. Torres-Zelada et al address this question in flies, where Gcn5 is part of distinct ATAC, SAGA/ADA, and CHAT complexes. These complexes incorporate different Ada2 subunits, with ATAC housing Ada2A and SAGA/ADA and CHAT housing different splice variants of Ada2b. The authors make use of these differences to genetically inactivate SAGA/ADA or CHAT in fly embryos, and then comparing changes in gene expression, histone acetylation and development. They also mutate the chiffon gene, which encodes two gene products, Db4 which regulates Cdc7, and Chiffon-B, which interacts with Gcn5 to nucleate formation of the CHAT complex. The authors find that almost all 2000+ genes regulated by SAGA/ADA are also regulated CHAT in fly embryos. They also identified 500+ genes that are uniquely regulated by CHAT. Surprisingly, they found that chiffon mutations that selectively disrupt CHAT functions have an even greater effects on histone H3 acetylation than do mutations in ada2b. These data suggest that chiffon/CHAT may influence the activity or targeting of multiple KATs beyond Gcn5. Overall, the studies presented here are well done, using a systematic approach, which judicious interpretation of data. The mechanisms by which CHAT vs SAGA are targeted (or not) to certain genes is not yet clear. Nor is it clear how CHAT influences global levels of H3 acetylation. Nonetheless, these studies constitute a significant step forward in understanding Gcn5 and CHAT functions.

1. **Reviewer #1:** Minor suggestion: early on, provide the reader with a small diagram indicating the differences in CHAT vs. SAGA/ADA composition.
   We thank the reviewer for this suggestion, and we have included a new schematic covering the differences in composition between the SAGA, ADA, and CHAT complexes. See revised Figure 1A and page 23, lines 677 – 678 for figure legend.

Reviewer #2:

In the manuscript by Torres-Zelada et al., the authors provide evidence that histone acetyltransferase (HAT) complexes, specifically, the Chiffon histone acetyltransferase (CHAT) complex, is critical for gene expression in the Drosophila early embryo. *Drosophila* contains

© 2021. Published by The Company of Biologists under the terms of the Creative Commons Attribution License (https://creativecommons.org/licenses/by/4.0/).

four Gcn5-containing complexes (SAGA, ATAC, CHAT and ADA) but it has been challenging to discern whether these HAT complexes support overlapping of distinct gene expression and/or have additional roles beyond regulating gene expression. Gcn5-binding protein Ada2b has two splice isoforms that are differentially acting; Ada2b-PB isoform is in SAGA; in contrast, Ada2b-PA associates with Gcn5, Ada3, Sgf29 and Chiffon, forming the CHAT complex. Chiffon is the Drosophila ortholog of Dbf4, which binds and activates the cell cycle kinase Cdc7 to initiate DNA replication. Therefore, by comparing/contrasting Ada2b-PA and Chiffon mutant phenotypes, the authors set out to determine the full function of the CHAT complex. Through this analysis, the authors conclude that a temporal switch in Chiffon function - supporting DDK/Cdc7 function prior to nc10/11 and then switching to support of CHAT subsequently. This model is supported by RNA-seq analysis that shows most of the Chiffon mutant gene expression changes are due to its support of HAT and not due to its connection to DDK; as well as the finding that the Chiffon-A product that binds Cdc7 is expressed early (NC3-NC14) as for Cdc7. The authors conclude that there is a switch at nc10/11 between the expression of Chiffon-A product that nucleates DDK formation and the Chiffon-B product that nucleates CHAT formation.

1. **Reviewer #2:** Experimental design and methods: In general, much more attention must be made to the description of experiments including attention to stage of embryos, sample size, as well as to quantitative and statistical analyses. This is a problem throughout the text but here are some representative examples of how important information was omitted:

   We apologize for the lack of experimental detail in the main text in the original submission. Much of this detail was previously included in the supplemental material, and we have now moved the methods in their entirety to the main text. We have also added text to the results and figure legends to clarify these details where appropriate. We hope this addresses your concerns and makes the manuscript clearer.

   i) The embryo collection timepoint used for the RNA-seq experiments is unstated; it is unclear what stage embryos are represented and how much variation would be expected.

   We thank the reviewer for this suggestion, and we have now included a more extensive description of each experiment, detailing embryo stages, number of biological replicates, and details about statistical analyses. We previously had many of those details in the Supplemental material, and we have now moved everything to the main text, adding also specific details as requested. Please see methods and figure legends with changes highlighted in yellow.

   ii) The method that was used for in situ quantification is not described within (e.g. Fig. S1C - nor was it included in figure legend).

   We thank the reviewer for this suggestion, and we have included the method that we have used to quantify images in Fig. S1. Please see method, page 17, lines 507 – 509.

   iii) In Fig S3A, the authors say that they observed significantly higher expression of the ΔN transgene, however, no statistical data is provided.

   We thank the reviewer for this suggestion, and we have added statistical analysis of the transgene expression in each genotype. These data are now included in Figure 3 as panel D.

   iv) In Fig. 3C, the authors say that 26 genes were differentially expressed between the 2 genotypes that they are describing, however, the shown data suggest that the number of genes might be smaller.

   We understand that it may have been difficult to identify those 26 genes based on the heatmap shown in Figure 3C. We now include a new Table S2 with a full list of the DEGs identified as chiffon-regulated (976) and gain-of-function genes (26).

© 2021. Published by The Company of Biologists under the terms of the Creative Commons Attribution License (https://creativecommons.org/licenses/by/4.0/).

v) At line 239, it is stated that Chiffon-A expression is observed as early as NC3. How were embryos staged? Identifying embryos from nc3-nc8 can be very challenging because nuclei have not all migrated to the periphery. In addition, the authors should costain Chiffon NHA-CFLAG with Cdc7.

We staged early embryos according to number and relative position of nuclei stained by DAPI. Although we present a single focal plane in the figure, we used multiple focal planes to stage embryos at the microscope. We have added details regarding staging in the methods. We have also replaced the image of a NC4 embryo in Figure 4B with a better image of a NC5 (16 nuclei) embryo. See revised Figure 4B.

We thank the reviewer for the suggestion to co-stain Cdc7 and Chiffon-A. This is a great idea, and we attempted to do this experiment by crossing the Cdc7-GFP flies (provided by Patrick O'Farrell) with our Chiffon-HA tagged flies, and immunostaining for GFP and HA. Unfortunately, we were not able to detect GFP expression in the progeny from this cross. We presume this is due to technical reasons, and the pattern of Chiffon-A (HA) staining that we observe in the early embryos strongly resembles the published expression pattern of Cdc7. We also have HA-tagged Cdc7 flies that we previously generated (Stephenson et al. JBC), but unfortunately that wouldn't allow us to distinguish between Chiffon and Cdc7. Currently, we do not have a suitable approach that would allow us to co-stain Cdc7 and Chiffon-A within the same embryo, but this is definitely something that we should pursue once we have suitable reagents.

vi) Lastly, many embryo images need to be replaced with healthy looking embryos in order to be able to make safe conclusions about gene expression. For example, in Fig 4D, nc6/7 DAPI staining of Ada2b-PB-FLAG tagged sample, the embryo looks unhealthy/dead.

We apologize for the unhealthy embryos that we inadvertently included in some of the previous figures. We have now replaced all unhealthy/dead embryos in Figure 4. Specifically, we have replaced NC10/11 (for Ada2b-PA) and NC6/7, NC10/11, and NC13 (Ada2b-PB). See revised Figure 4.

2. **Reviewer #2:** Embryos staging: The lack of H3K14ac staining of embryos at stage 13 (figure1B) is convincing. On the other hand, gene expression in the early embryo is very dynamic and the embryo stage of samples is unclear (e.g. Fig. 1C-E). More careful description of the staging is required; it is hard to evaluate the study properly without this information. Furthermore, as mentioned above, the embryos in Figure 4B and D need to be replaced with healthy, stage appropriate ones. The stage of the embryos used in each case should be clearly stated either in the main text or in each figure's caption.
We thank the reviewer for this comment and we have added a more careful description of embryo stages per experiment in the appropriate figure legend, method section and text (see also comment #1.1).

3. **Reviewer #2:** Additional references: The authors should place their study in context of the current field. Several studies have examined the role of other ubiquitously expressed timing factors. Are there any timing factors that might relate, specifically, to CHAT action proposed by the authors to initiate at nc10/11?

We now discuss the role of other timing factors, with a particular focus on Zelda, in the new Discussion Section in the revised manuscript. See Discussion Section, page 12, lines 372 – 393.

4. **Reviewer #2:** Data interpretation: In Fig. S2D, the authors argue for global decrease in histone acetylation (not only at CHAT-regulated genes). How can specific targets of CHAT then be identified?

We agree that it is critical to identify direct targets of CHAT and of Chiffon-B (in case Chiffon-B has additional functions outside of CHAT). The best approach for this would

© 2021. Published by The Company of Biologists under the terms of the Creative Commons Attribution License (https://creativecommons.org/licenses/by/4.0/).

be genome-wide mapping of Chiffon-B using a technique such as ChIP-seq, and we have made several attempts to do this over the past year. Unfortunately, we have had technical difficulties obtaining reproducible ChIP-seq profiles for CHAT, potentially due to the presence of only a single FLAG tag on the Chiffon-FL transgene, reducing IP efficiency. We are in the process of generating 3xFLAG tagged Chiffon-FL to try and obtain better quality ChIP-seq data, and we hope that this approach will be more successful. We address this point in the Discussion, page 12, lines 365 – 371.

5. Reviewer #2: The GEO data should be available to the reviewers for evaluation during the review process. Please provide the reviewers' with a token for access.

   The GEO number is provided in the methods section. See page 18, lines 536 – 538.The reviewer code access is upgvguwidnqbnof.

6. **Reviewer #2:** Additional support for the model that Chiffon supports entry of Ada2b-PB into the nucleus. In the DN Chiffon mutant, does HA-Ada2-PA enter the nucleus at an earlier stage?

   We thank the reviewer for this suggestion and we agree that this remains an important and unanswered question from this study. Unfortunately, we do not have the reagents available to do this experiment because our chiffon mutant flies that express Chiffon-N (WT DDK; null CHAT) have the Chiffon-N transgene tagged with FLAG and HA, as do the Ada2b-PA and PB transgenes. We do not therefore have a system in which we could distinguish the Ada2-PA or PB immunostaining signal from Chiffon. We would like to generate untagged rescue flies to be able to perform this or similar experiments in the future, but this would be beyond the scope and timing of the current study.

7. **Reviewer #2:** What is known about the ability of Chiffon to regulate H3K9ac levels? If this has been investigated please add to the intro and/or discussion.

   We thank the reviewer for this comment. The CHAT complex specifically regulates acetylation of histone H3 at lysine 9, 14, and 18 in ovary follicle cells (Torres-Zelada et al. 2019). We have added these details in the revised manuscript. See text, page 6, lines 148 – 150.

8. **Reviewer #2:** Line 265: Typo “nucleusnuclei” should be fixed.

   We have fixed this error in the revised text, page 11 line 315.

9. **Reviewer #2:** Line 276: As stated by the authors, CHAT formation occurs just prior to the de novo large-scale recruitment of RNA pol II that leads to activation of the zygotic genome. It would be helpful to relate CHAT function to that of pioneer factors, Zelda, Odd paired, etc. Could HAT complexes relate to differential action of the multiple pioneer factors acting in the early embryo?

   Yes, we agree this is a potential function for CHAT and other HAT complexes, and we now discuss this point in the Discussion Section. See page 12 lines 372 - 393.

10. **Reviewer #2:** Line 288: The supporting literature for the 2 waves of transcriptional activation should be cited in the paper.

    We have added this reference. See page 13, line 397.

11. **Reviewer #2:** Figure 2G. The scale of the Y axis is missing as well as information regarding the browser that was used to support this analysis.
    We have added the y axis scale and information regarding the browser (IGV) to legend and methods. We have also moved the snapshots of the unique H3K14ac peaks to the main Figure 2. See revised Figure 2.

© 2021. Published by The Company of Biologists under the terms of the Creative Commons Attribution License (https://creativecommons.org/licenses/by/4.0/).

12. **Reviewer #2:** Figure 4D. Panel Ada2b-PB-FLAG DAPI: the embryo is unhealthy (potentially dead).

    We have replaced all unhealthy/dead embryos in Figure 4. We apologize for the inclusion of the dead embryo in the previous version!

13. **Reviewer #2:** Figure S4B. The authors should use standard methods to support embryo staging and present their data accordingly. This could include costaining with genes that initiate expression at particular stages and/or using a quantitative approach to calculate nuclei per area and extrapolate to nc stage.

    We apologize for the confusion regarding Figure S4 (which is now Figure 5 in the revised version). These data were not based on immunostaining, but rather on qRT- PCR. For this experiment, we wanted to test if there were differences in the relative abundance of the mRNA regions corresponding to Chiffon-A and Chiffon-B. If we had observed differences, this would have suggested that alternative splicing could have explained the switch in protein expression that we observe by immunostaining. To do this experiment, we collected early stage embryos in 3 time windows (0 – 1h, 1 – 2h, 2 – 3h) and then extracted RNA from each single embryo. Thus, embryos were not staged (except broadly in terms of time windows) for this experiment. Instead, we examined relative expression of two genes that are known to be expressed either early or later in these stages: nanos and even skipped. We then ranked embryos by nanos/eve expression, but this was only to give a relative idea of staging. Instead, the important question in this figure was whether there were any differences in Chiffon-A versus Chiffon-B mRNA levels within a single embryo.

    We apologize if this was not clear in the text and we have added more details about this experiment in the results and figure legend. See page 10, lines 299 – 311.

14. DDK should be explained in the context of the abstract. To a non-expert, its significance is unclear.

    We have added a concise description of DDK in both the Abstract and Introduction. See Abstract, page 2, lines 24 – 27 and Introduction page 3 lines 56 – 59.

Reviewer #3:

Torres-Zelada et al have compared Ada2b mutants to chiffon mutants in Drosophila with respect to gene expression changes (RNA-seq) and histone acetylation (H3K14ac ChIP- seq). Ada2b mutants disrupt three histone acetyltransferase (HAT) complexes in flies, SAGA, ADA and CHAT. Using rescue transgenes expressing either the PA or PB splice isoforms, the authors identify redundant, cooperative and genes unique to SAGA/ADA or CHAT. Although SAGA/ADA/CHAT are redundant for expression of most genes that are deregulated in Ada2b mutants, CHAT has a stronger effect and controls more uniquely regulated genes. Surprisingly, chiffon mutants that disrupt CHAT but not SAGA/ADA has a stronger effect on H3K14ac than Ada2b mutants that inactive all three complexes, and the overlap in gene expression changes between chiffon and Ada2b mutants is poor. The chiffon locus is complicated, since it may be regulated translationally. The authors nicely demonstrate that the chiffon N-terminus that interacts with Cdc7 is required during the early embryonic nuclear cell cycles, whereas the C-terminus that forms CHAT takes over at later embryonic stages. This is important work that uses genetic analyses to decipher the function of HAT complexes in vivo, but the results are not that easy to interpret. The use of different alleles in trans, the many replicates for RNA-seq, and spike-in normalized ChIP-seq ensures high quality data, but the immunostainings and imaging needs to be improved. At present, the conclusions from these stainings are not supported by the images.

1. **Reviewer #3**: The paper would benefit from a more clear description of the experimental setup. It took me some time to realize that the Ada2b rescue transgenes were driven by endogenous promoters and not by UAS sites. Fig. 1A could illustrate this in a better way.

© 2021. Published by The Company of Biologists under the terms of the Creative Commons Attribution License (https://creativecommons.org/licenses/by/4.0/).

We thank the reviewer for this comment and Reviewer #2 raised a similar point (comment #1). We have added more details about the experiments to the methods, figure legends, and results text. We have also included more details about the rescue constructs for both the Chiffon and Ada2b transgenes in the methods, results and figure legends, and show schematics illustrating these transgenes in the respective figures. These transgenes were previously published, but we have repeated the information briefly in this manuscript to make it easier for readers to understand what these constructs contain. See page 15, lines 422 – 428.

2. **Reviewer #3:** I could not find information on what embryonic stage was used for RNA-seq in Ada2b mutants.

   We now include information about the embryo stages used for all experiments in the legend, methods and results text. See changes highlighted in yellow.

3. **Reviewer #3:** The quality of the immunostainings and the resulting images need improvement. H3K14ac has previously been shown to be strongest in the CNS, but this is not clear in Fig. 1B and Fig. S1 (Fig. 2B looks better).

   We thank the reviewer for this comment and we have updated both Figures 1B and S1 with better images for H3K14ac in ada2b mutants.

4. **Reviewer #3:** Figure 4 is even more difficult to interpret. Is the signal from Chiffon-C Ab in NC4 background fluorescence? Did the authors test the specificity of this Ab on chiffon mutants?.

   We apologize if this was unclear - yes, the signal from the Chiffon-C antibody in NC4 embryos represents background fluorescence because we do not detect Chiffon-B (CHAT, visualized using the Chiffon C terminal antibody) in this stage. We have tested the specificity of this Chiffon C-terminal antibody for immunostaining using chiffon mutant embryos (Figure 2B). We now explain this more clearly in the text. See page 10, lines 284 – 286.

5. **Reviewer #3:** What is signal and what is background using the HA and FLAG Abs in Fig. 4B and D? The HA signal in Fig. S4A looks like gut autofluorescence.

   We apologize again if this wasn't clear in the original submission. First, the HA signal in Fig S4A indeed represents autofluorescence because these embryos (w1118) do not have any HA-tagged transgene. In Fig 4B and D, the HA and FLAG signals that colocalize with the DAPI foci, representing nuclei, are the real Chiffon-A or Ada2b-PA/PB immunostaining signal. We show background immunostaining for HA and FLAG antibodies in Fig. S5 for comparison. These images were not taken on the confocal microscope, likely accounting for the higher background signal.

6. **Reviewer #3:** The imaging parameters should be described in the Methods.

   We now describe the imaging parameters and analysis fully in the methods section. See page 17, lines 507 – 509.

7. **Reviewer #3:** The stronger effect of chiffon than of Ada2b mutants on H3K14ac is perplexing. Since the authors use two alleles in trans and have previously shown that H3K14ac levels in chiffon mutants can be rescued by chiffon transgenes, this is most likely not caused by second site mutations. The authors should expand their discussion on what could be causing this phenotype.

   We have added a new Discussion section that addresses this point. We agree this is a strange observation and is not something we expected to see given our previous findings in ovary follicle cells. See page 12, lines 346 – 371.

© 2021. Published by The Company of Biologists under the terms of the Creative Commons Attribution License (https://creativecommons.org/licenses/by/4.0/).

8. **Reviewer #3:** What could be the explanation of the poor overlap of gene expression changes in chiffon and Adab2 mutants? Is there anything from the previous mass spectrometry experiments performed by the authors that indicate a function for Chiffon outside the CHAT and DDK complexes?

   Similar to our response to comment #7, we suspect additional roles for Chiffon-B outside of the CHAT complex in embryos. We searched our previous mass spectrometry data from S2 cells for any potential Chiffon-B interactors such as other HATs, but these were not present. However, it might be that any Chiffon-B interactions are only present in embryos. This is an interesting direction for our future experiments. See Discussion page 12.

9. **Reviewer #3:** Given that Chiffon could have other functions not related to CHAT or DDK, the title of the paper should be changed.

   We thank the reviewer for this suggestion and we have changed the title of the manuscript to "Chiffon triggers global histone H3 acetylation and expression of developmental genes in Drosophila embryos".

10. **Reviewer #3:** Figures 2E-G are not mentioned in the text.

    We have fixed this error.

Second decision letter

MS ID#: JOCES/2021/259132

MS TITLE: Chiffon triggers global histone H3 acetylation and expression of developmental genes in Drosophila embryos

AUTHORS: Eliana F. Torres-Zelada, Smitha George, Hannah R. Blum, and Vikki Marie Weake
ARTICLE TYPE: Research Article

We have now reached a decision on the above manuscript.

To see the reviewers' reports and a copy of this decision letter, please go to: https://submit-jcs.biologists.org and click on the 'Manuscripts with Decisions' queue in the Author Area. (Corresponding author only has access to reviews.)

As you will see, the reviewers gave favourable reports but raised some critical points that will require amendments to your manuscript. I hope that you will be able to carry these out because I would like to be able to accept your paper, depending on further comments from reviewers.

We are aware that you may be experiencing disruption to the normal running of your lab that makes experimental revisions challenging. If it would be helpful, we encourage you to contact us to discuss your revision in greater detail. Please send us a point-by-point response indicating where you are able to address concerns raised (either experimentally or by changes to the text) and where you will not be able to do so within the normal timeframe of a revision. We will then provide further guidance. Please also note that we are happy to extend revision timeframes as necessary.

Please ensure that you clearly highlight all changes made in the revised manuscript. Please avoid using 'Tracked changes' in Word files as these are lost in PDF conversion.

I should be grateful if you would also provide a point-by-point response detailing how you have dealt with the points raised by the reviewers in the 'Response to Reviewers' box. Please attend to

© 2021. Published by The Company of Biologists under the terms of the Creative Commons Attribution License (https://creativecommons.org/licenses/by/4.0/).

all of the reviewers' comments. If you do not agree with any of their criticisms or suggestions please explain clearly why this is so.

Reviewer 1

Advance summary and potential significance to field

This revised paper is even stronger and more clear with the additional discussion of experimental details suggested by the other reviewers. This work provides important insights to the division of labor between Gcn5 containing KAT complexes during fly development and will be of interest to a broad readership.

Comments for the author

The authors have carefully and thoroughly addressed all previous criticisms and suggestions.

Reviewer 2

Advance summary and potential significance to field

The authors conclude that a temporal switch in Chiffon function - supporting DDK/Cdc7 function prior to nc10/11 and then switching to support of CHAT subsequently. This model is supported by RNA-seq analysis that shows most of the Chiffon mutant gene expression changes are due to its support of HAT and not due to its connection to DDK; as well as the finding that the Chiffon-A product that binds Cdc7 is expressed early (NC3-NC14) as for Cdc7. The authors conclude that there is a switch at nc10/11 between the expression of Chiffon-A product that nucleates DDK formation and the Chiffon-B product that nucleates CHAT formation.
The data are of interest to the field and support the view that Chiffon has dual function.

Comments for the author

The manuscript is much approved. The additions have greatly improved the clarity of the presentation. One important control is missing regarding specificity of the Chiffon-B antibody (see point 2) and these data are required because otherwise conclusions about Chiffon-A/B's relative expression (Fig. 4) do not stand. A few other minor suggestions are noted, in addition.

1. Fig 5: in order to interpret the data, the reader has to understand that the HA tag monitors chiffonA and the FLAG tag monitors chiffonB. Can information regarding this be added to the schematic showing position of the qPCR primers.
It's quite challenging to follow all the isoforms…

2. The antibody stated to be specific for Chiffon-B needs to be further tested for specificity during the early embryonic stages (i.e. nc4-14) when the authors are making conclusions about Chiffon isoform roles in managing the MAT. Embryos at stage 9/13 (Fig. 2B) are not the appropriate negative control for the data presented in Fig. 4 (nc4-14). The authors are able to generate germline clones for chiffon as part of Fig. 6. The Chiffon C-term antibody specific for Chiffon-B should be used to stain all the genotypes displayed in Fig. 6A. This would provide strong support that the antibody is good, and is the only way that the data in Fig. 4 can be trusted.

3. Figure 4 legend, line 724: please provide reference for "Pol II recruitment occurs in NC13"…do the authors mean "widespread Pol II recruitment occurs…"?

4. Fig. 5A: embryos at relative timepoint positions 9 and 10 have high nanos and low/intermediate eve – how were these classified? Are they in positions 9 and 10 because they were the embryos with highest "eve" within the assayed cohort of 10? Please note groups of 10 with shading and/or notation in figure legend. The Methods does not give sufficient details.

© 2021. Published by The Company of Biologists under the terms of the Creative Commons Attribution License (https://creativecommons.org/licenses/by/4.0/).

5. I spent a long time trying to understand Fig. S2 and how it demonstrates that Ada2b-PA is "required and sufficient" for complex formation. Please explain.

6. Fig 4 – how many embryos were assayed for each timepoint (e.g. nc4)? Were these images captured under identical settings? Better yet would be to quantify levels but at minimum please include how many embryos were assayed. I don't see this information in the figure legend or the methods section labelled "Immunostaining".

Reviewer 3

Advance summary and potential significance to field

In their revised manuscript, Torres-Zelada et al have addressed all of my major concerns.

Comments for the author

The conditions used for imaging are still not clear. In the Methods, they write that they have used a Leica microscope, but not what model and how the images were processed. Are the embryo images shown optical sections or projections from multiple sections?

Second revision

Author response to reviewers' comments

RESPONSE TO REVIEWERS

We would like to thank the reviewers for their comments and suggestions, and we are glad that the revised manuscript was clearer and easier to follow. Changes are marked in the revised text in yellow, and we provide an updated version of Figure 5. There are no changes to any other figures or supplementary material. Reviewer #1 did not have any additional comments, so we only provide responses to Reviewers #2 and #3. We also include the relevant text changes in red below the response to each comment.

Reviewer #2:
Comment #1: Fig 5: in order to interpret the data, the reader has to understand that the HA tag monitors chiffonA and the FLAG tag monitors chiffonB. Can information regarding this be added to the schematic showing position of the qPCR primers. It's quite challenging to follow all the isoforms…

We have added Chiffon-A and Chiffon-B tags to this schematic and to the graph to make this figure (hopefully) easier to follow. We agree that following the complicated gene structure and isoforms for Chiffon is a challenge! See revised Figure 5.

Comment #2: The antibody stated to be specific for Chiffon-B needs to be further tested for specificity during the early embryonic stages (i.e. nc4-14) when the authors are making conclusions about Chiffon isoform roles in managing the MAT. Embryos at stage 9/13 (Fig. 2B) are not the appropriate negative control for the data presented in Fig. 4 (nc4-14). The authors are able to generate germline clones for chiffon as part of Fig. 6. The Chiffon C-term antibody specific for Chiffon-B should be used to stain all the genotypes displayed in Fig. 6A. This would provide strong support that the antibody is good, and is the only way that the data in Fig. 4 can be trusted.

We thank the reviewer for this suggestion, but we do not agree that this experiment is necessary to validate the specificity of the Chiffon antibody because we already show that this antibody is specific for immunostaining embryos in Figure 2B. In addition, because Chiffon-B is not expressed in early embryos (only Chiffon-A is expressed prior to NC10/11) – characterizing the specificity of the antibody at this stage would not be possible. I am further concerned that using chiffon

© 2021. Published by The Company of Biologists under the terms of the Creative Commons Attribution License (https://creativecommons.org/licenses/by/4.0/).

germline clones as a negative control for testing the specificity of the antibody would be problematic since these are dying fairly early on in development.

Comment #3: Figure 4 legend, line 724: please provide reference for “Pol II recruitment occurs in NC13”…do the authors mean “widespread Pol II recruitment occurs…”?

We apologize – this sentence was not written very clearly. We have now replaced that sentence in the Figure 4 legend with: “There is an initial wave of Pol II recruitment and zygotic transcription at NC8 followed by a second more widespread wave of Pol II recruitment and transcription at NC13.” Please see page 24, line 729.

Comment #4: Fig. 5A: embryos at relative timepoint positions 9 and 10 have high nanos and low/intermediate eve – how were these classified? Are they in positions 9 and 10 because they were the embryos with highest “eve” within the assayed cohort of 10? Please note groups of 10 with shading and/or notation in figure legend. The Methods does not give sufficient details.

These are all single embryos – not groups of 10. Embryos are ranked by the ratio of eve/nanos expression just to provide some indication of whether these are “early” or “late”. The main point of this analysis was to ask if there are any differences in the relative transcript level for the Chiffon-A and Chiffon-B regions of the chiffon transcript i.e. is there any indication of alternative splicing. Consistent with published RNA-seq data and our own data, we do not observe any evidence of alternative splicing for chiffon that could generate Chiffon-A and Chiffon-B proteins. We have added some additional text to the relevant results section and this figure legend to try and make this analysis clearer: see page 24, line 742 and page 11, line 310.

page 24, line 742: “Transcript levels were measured in single embryos and are shown relative to Rpl32. Single embryos were ranked using the ratio of expression of nanos (nos, early) versus even skipped (eve, late) to provide a relative indication of early versus later developmental stage.”

page 11, line 310: “To provide a relative indication of stage, we ranked single embryos by the ratio of expression of nanos (nos) and even skipped (eve), which are expressed early or late during the nuclear cycles, respectively (Thomsen et al., 2010).”

Comment #5: I spent a long time trying to understand Fig. S2 and how it demonstrates that Ada2b-PA is “required and sufficient” for complex formation. Please explain.

We apologize if this was unclear, and we now expand our description of these data in the text to explain this point. Briefly, Gcn5 requires other proteins for its ability to acetylate nucleosomal substrates. We show that Ada2b-PA binds directly to Gcn5 and Ada3 using recombinant proteins expressed in Baculovirus, and that Ada3 binds Sgf29. Thus, in the absence of Chiffon – Ada2b-PA can interact with Gcn5, Ada3, and Sgf29 forming the minimal complex that should be required for Gcn5 nucleosomal HAT activity. However, it is unclear how such a complex would be targeted to chromatin - and presumably Chiffon has some function in this. Despite this, the recombinant protein studies indicate that in the absence of Ada2b-PA, the rest of the CHAT complex should not assemble. Thus, it is particularly puzzling that ada2b embryos show less of a decrease in H3K14ac relative to chiffon embryos. See page 6, line 163.

“Because Ada2 subunits are essential for the nucleosomal HAT activity of Gcn5 (Grant et al., 1997), we next asked if Ada2b-PA, Gcn5, Sgf29, and Ada3 could associate in the absence of Chiffon. To do this, we expressed each subunit in Sf21 cell using Baculovirus, and tested for direct interaction by co-immunoprecipitation (Fig S2). Using this approach, we found that Ada2b-PA directly binds Gcn5 and Ada3, but not Sgf29, which is recruited instead through Ada3. Because Ada2b-PA is necessary and sufficient for Gcn5, Sgf29 and Ada3 to associate even in the absence of Chiffon, we would therefore expect that loss of ada2b would disrupt CHAT formation. Thus, the loss of Chiffon in embryos has a stronger impact on histone acetylation that can be explained simply by loss of SAGA, ADA, and CHAT HAT activity. Moreover, the decrease in H3K14ac in chiffon embryos differs from our previous observations in ovary follicle cells in which chiffon and ada2b mutants both showed ~50% decreases in H3K14ac relative to their respective controls (Torres-Zelada et al., 2019).”

© 2021. Published by The Company of Biologists under the terms of the Creative Commons Attribution License (https://creativecommons.org/licenses/by/4.0/).

Comment #6: Fig 4 – how many embryos were assayed for each timepoint (e.g. nc4)? Were these images captured under identical settings? Better yet would be to quantify levels but at minimum please include how many embryos were assayed. I don't see this information in the figure legend or the methods section labelled "Immunostaining".

We imaged at least 3 embryos for each timepoint/genotype and provide representative images in Figure 4. This information is now included in the figure legend for Figure 4 and in the methods, page 24 line 733 and page 17, line 515.

page 24 line 733: "Representative images are shown (n ≥ 3)."

page 17, line 515: "Multiple sections were examined for each embryo (n ≥ 3), and single optical sections are shown for each representative image."

Reviewer #3:

Comment #1: The conditions used for imaging are still not clear. In the Methods, they write that they have used a Leica microscope, but not what model and how the images were processed. Are the embryo images shown optical sections or projections from multiple sections?

We apologize for leaving out the model number in the revised text. We now describe the microscope model and imaging conditions in the methods section. Yes, we used optical sections for this analysis. See page 17, line 512.

"Images were taken using a Leica DM6B fluorescent microscope equipped with CTR6-LED and DFC450 digital camera. Acetylation levels were determined as average sum intensity values for fluorescence comparing GFP (mutant embryo) with the non-GFP embryo (WT homozygous sibling) using Image J software. Multiple sections were examined for each embryo (n ≥ 3), and single optical sections are shown for each representative image."

Third decision letter

MS ID#: JOCES/2021/259132

MS TITLE: Chiffon triggers global histone H3 acetylation and expression of developmental genes in Drosophila embryos

AUTHORS: Eliana F. Torres-Zelada, Smitha George, Hannah R. Blum, and Vikki Marie Weake
ARTICLE TYPE: Research Article

I am happy to tell you that your manuscript has been accepted for publication in Journal of Cell Science, pending standard ethics checks.

© 2021. Published by The Company of Biologists under the terms of the Creative Commons Attribution License (https://creativecommons.org/licenses/by/4.0/).
